# Methodological Framework for World Health Organization Estimates of the Global Burden of Foodborne Disease

**DOI:** 10.1371/journal.pone.0142498

**Published:** 2015-12-03

**Authors:** Brecht Devleesschauwer, Juanita A. Haagsma, Frederick J. Angulo, David C. Bellinger, Dana Cole, Dörte Döpfer, Aamir Fazil, Eric M. Fèvre, Herman J. Gibb, Tine Hald, Martyn D. Kirk, Robin J. Lake, Charline Maertens de Noordhout, Colin D. Mathers, Scott A. McDonald, Sara M. Pires, Niko Speybroeck, M. Kate Thomas, Paul R. Torgerson, Felicia Wu, Arie H. Havelaar, Nicolas Praet

**Affiliations:** 1 Department of Virology, Parasitology and Immunology, Faculty of Veterinary Medicine, Ghent University, Merelbeke, Belgium; 2 Insitute of Health and Society (IRSS), Université catholique de Louvain, Brussels, Belgium; 3 Department of Biomedical Sciences, Institute of Tropical Medicine, Antwerp, Belgium; 4 Department of Animal Sciences and Emerging Pathogens Institute, University of Florida, Gainesville, FL, United States of America; 5 Department of Public Health, Erasmus University Medical Center, Rotterdam, The Netherlands; 6 Centers for Disease Control and Prevention, Atlanta, GA, United States of America; 7 Department of Neurology, Boston Children's Hospital, Harvard Medical School, Boston, MA, United States of America; 8 Department of Environmental Health, Harvard School of Public Health, Boston, MA, United States of America; 9 Department of Medical Sciences, School of Veterinary Medicine, University of Wisconsin in Madison, Wisconsin, United States of America; 10 Public Health Agency of Canada, Guelph, Ontario, Canada; 11 Institute of Infection and Global Health, University of Liverpool, Leahurst Campus, Neston, United Kingdom; 12 International Livestock Research Institute, Nairobi, Kenya; 13 Gibb Epidemiology Consulting, Arlington, VA, United States of America; 14 National Food Institute, Danish Technical University, Lyngby, Denmark; 15 National Centre for Epidemiology and Population Health, Research School of Population Health, The Australian National University, Canberra, Australia; 16 Institute of Environmental Science and Research, Christchurch, New Zealand; 17 Department of Health Statistics and Information Systems, World Health Organization, Geneva, Switzerland; 18 Centre for Infectious Disease Control, National Institute for Public Health and the Environment (RIVM), Bilthoven, The Netherlands; 19 Section of Epidemiology, Vetsuisse Faculty, University of Zurich, Zurich, Switzerland; 20 Department of Food Science and Human Nutrition, Department of Agricultural, Food, and Resource Economics, Michigan State University, East Lansing, MI, United States of America; 21 National Institute for Public Health and the Environment (RIVM), Bilthoven, the Netherlands; 22 Utrecht University, Utrecht, Netherlands; The National Institute for Public Health and the Environment, NETHERLANDS

## Abstract

**Background:**

The Foodborne Disease Burden Epidemiology Reference Group (FERG) was established in 2007 by the World Health Organization to estimate the global burden of foodborne diseases (FBDs). This paper describes the methodological framework developed by FERG's Computational Task Force to transform epidemiological information into FBD burden estimates.

**Methods and Findings:**

The global and regional burden of 31 FBDs was quantified, along with limited estimates for 5 other FBDs, using Disability-Adjusted Life Years in a hazard- and incidence-based approach. To accomplish this task, the following workflow was defined: outline of disease models and collection of epidemiological data; design and completion of a database template; development of an imputation model; identification of disability weights; probabilistic burden assessment; and estimating the proportion of the disease burden by each hazard that is attributable to exposure by food (i.e., source attribution). All computations were performed in R and the different functions were compiled in the R package 'FERG'. Traceability and transparency were ensured by sharing results and methods in an interactive way with all FERG members throughout the process.

**Conclusions:**

We developed a comprehensive framework for estimating the global burden of FBDs, in which methodological simplicity and transparency were key elements. All the tools developed have been made available and can be translated into a user-friendly national toolkit for studying and monitoring food safety at the local level.

## Introduction

The Foodborne Disease Burden Epidemiology Reference Group (FERG) was established in 2007 by the World Health Organization (WHO) to estimate the global burden of foodborne diseases (FBDs) [1]. In 2012, FERG established a Computational Task Force (CTF) to derive FBD burden estimates using epidemiological information generated by the hazard-based and source attribution task forces [2]. The aim of this paper is to describe the methodological framework developed by the CTF for this task.

Variation in methodological choices has been identified both in general and in foodborne-specific burden of disease studies, impeding comparability of burden estimates across studies [3,4]. To ensure accuracy, utility and comparability with other existing health metric initiatives, FERG decided to quantify the burden of FBDs in terms of Disability-Adjusted Life Years (DALYs), a health gap measure expressing the number of healthy life years lost due to reduction of health and death. Further methodological choices were discussed during the fourth formal meeting of FERG in 2010 [5], and confirmed at the FERG Strategic Planning Meeting in 2011 and at the fifth FERG meeting in 2013 [6,7]. The main methodological decision was that DALYs were to be calculated in a hazard- and incidence-based approach [8].

Building on the FERG approach, the CTF tasks were (1) to develop the necessary tools for DALY calculation and (2) to implement these tools for estimating the burden of 36 FBDs, including 21 enteric diseases caused by bacteria, viruses and protozoa, 11 non-enteric parasitic diseases, and 4 diseases caused by chemicals and toxins.

In this paper, we (1) describe the approach taken for quantifying the burden of FBD; (2) describe the steps and methodological choices to calculate DALYs resulting from FBDs; (3) describe traceability and transparency during the process; and (4) discuss limitations and future directions. All tools that were developed by the CTF are available as Supporting Information, allowing the reader to access, explore and use these different tools.

## Hazard- and Incidence-Based DALY Approach

Strong and reliable burden of disease estimates are crucial for setting priorities in public health and biomedical research [9,10]. Ranking disease impact may be based on disease occurrence (prevalence or incidence) or on the number of deaths (mortality). However, these simple measures of population health do not provide a full picture of the impact of specific diseases on human health. Indeed, while certain diseases may be very common, their clinical impact may be limited. Infection with a highly prevalent parasite such as *Enterobius vermicularis* (also known as pinworm or threadworm) for instance has a very low burden because most of the cases are mild to asymptomatic and self-limiting [11]. Likewise, ignoring the age at which people die, and thus not considering how many years of healthy life might be lost due to a premature death does not fairly capture the impact of mortality. Disease severity, defined by the health impact and duration of the concerned symptoms and the life expectancy at the age of death, should thus also be taken into account when quantifying burden of disease. Furthermore, simple measures of population health do not combine the impacts of morbidity and mortality. This prohibits a comparative ranking of highly morbid, but not necessarily fatal diseases such as chorioretinitis and highly lethal diseases such as liver cancer, complicating decisions on resource allocation priorities.

To overcome the limitations of simple measures of population health, summary measures of population health (SMPHs) have been developed as an additional source of information for measuring disease burden. Among these SMPHs, the Disability-Adjusted Life Year (DALY) is currently the most widely used in public health research. Originally developed to quantify and compare the burden of diseases, injuries and risk factors across countries, the DALY summarizes the occurrence and impact of morbidity and mortality in a single measure [12,13]. The DALY is the key measure in the Global Burden of Disease (GBD) studies and is officially adopted by WHO for reporting on health information [14,15].

The DALY is a health gap measure. It measures the healthy life years lost due to a disease or injury. DALYs are calculated by adding the adjusted number of years lived with disability (YLDs) and the number of years of life lost due to premature mortality (YLLs):

YLD = Number of incident cases x Duration until remission or death x Disability Weight

YLL = Number of deaths x Residual life expectancy at the age of death

Different approaches can be taken for calculating DALYs, depending on whether the interest lies in quantifying the burden of a health outcome (such as diarrhea), a hazard (as defined by the Codex Alimentarius Commission, e.g. a biological agent that may cause illness in humans such as *Salmonella enterica*), or a risk factor (e.g. an exposure that increases the likelihood of illness such as unsafe water) [16]. Since FERG is concerned with the burden of FBDs, which are caused by a wide range of hazards (bacteria, viruses, parasites, chemicals, and toxins), a natural choice is the hazard-based approach. This approach defines the burden of a specific foodborne hazard as that resulting from the health states, i.e., symptoms and sequelae, including death, that are causally related to the concerned hazard transmitted through food, and which may become manifest at different time scales or have different severity levels [8]. This approach thus allows for a comprehensive estimate of the burden of disease due to a certain hazard, including sequelae, which may have a higher burden than acute illness alone [17–19]. Table 1 shows the hazards and related health states that were included in FERG's global burden of FBD estimates.

**Table 1:** FERG hazards, causally related health states and corresponding disability weights (DWs). Details on the derivation of the DWs are provided in S2 Table.

Hazard	Health state	DW
**Diarrheal disease agents**		
Norovirus	Diarrheal disease ^a^	0.074
*Campylobacter* spp.	Diarrheal disease ^a^	0.101
	Guillain-Barré syndrome	0.445
Enteropathogenic *E*. *coli*	Diarrheal disease ^a^	0.074
Enterotoxigenic *E*. *coli*	Diarrheal disease ^a^	0.074
Shiga toxin-producing *E*. *coli*	Diarrheal disease ^a^	0.091
	Hemolytic uremic syndrome	0.210
	End-stage renal disease	0.573
Non-typhoidal *S*. *enterica*	Diarrheal disease ^a^	0.101
	Invasive salmonellosis	0.210
*Shigella* spp.	Diarrheal disease ^a^	0.101
*Vibrio cholerae*	Diarrheal disease ^a^	0.194
*Cryptosporidium* spp.	Diarrheal disease ^a^	0.074
*Entamoeba histolytica*	Diarrheal disease ^a^	0.074
*Giardia* spp.	Diarrheal disease ^a^	0.074
**Invasive infectious disease agents**		
Hepatitis A Virus	Hepatitis	0.108
*Brucella* spp.	Acute brucellosis	0.108
	Chronic brucellosis	0.079
	Orchitis	0.097
*Listeria monocytogenes*, perinatal	Sepsis	0.210
	Central nervous system infection	0.426
	Neurological sequelae	0.292
*Listeria monocytogenes*, acquired	Sepsis	0.210
	Central nervous system infection	0.426
	Neurological sequelae	0.292
*Mycobacterium bovis*	Tuberculosis	0.331
*Salmonella* Paratyphi	Paratyphoid fever	0.210
	Liver abscesses and cysts	0.254
*Salmonella* Typhi	Typhoid fever	0.210
	Liver abscesses and cysts	0.254
*Toxoplasma gondii*, congenital	Intracranial calcification	0.010
	Hydrocephalus	0.360
	Chorioretinitis, early in life	0.033
	Chorioretinitis, later in life	0.033
	CNS abnormalities	0.360
*Toxoplasma gondii*, acquired	Chorioretinitis, mild	0.004
	Chorioretinitis, moderate	0.033
	Chorioretinitis, severe	0.191
	Acute illness	0.053
	Post-acute illness	0.254
**Enteric intoxications**		
*Bacillus cereus* ^b^	Acute intoxication	0.061
*Clostridium botulinum* ^b^	Moderate/mild botulism	0.198
	Severe botulism	0.445
*Clostridium perfringens* ^b^	Acute intoxication	0.061
*Staphylococcus aureus* ^b^	Acute intoxication	0.061
**Cestodes**		
*Echinococcus granulosus*, cases seeking treatment	Pulmonary cystic echinococcosis	0.192
	Hepatic cystic echinococcosis	0.123
	Central nervous system cystic echinococcosis	0.221
*Echinococcus granulosus*, cases not seeking treatment	Pulmonary cystic echinococcosis	0.015
	Hepatic cystic echinococcosis	0.012
	Central nervous system cystic echinococcosis	0.054
*Echinococcus multilocularis*	Alveolar echinococcosis	0.123
*Taenia solium*	Epilepsy: treated, seizure free	0.072
	Epilepsy: treated, with recent seizures	0.319
	Epilepsy: severe	0.657
	Epilepsy: untreated	0.420
**Nematodes**		
*Ascaris* spp.	Ascariasis infestation	0.030
	Mild abdominopelvic problems due to ascariasis	0.012
	Severe wasting due to ascariasis	0.127
*Trichinella* spp.	Acute clinical trichinellosis	0.637
**Trematodes**		
*Clonorchis sinensis*	Abdominopelvic problems due to heavy clonorchiosis	0.123
*Fasciola* spp.	Abdominopelvic problems due to heavy fasciolosis	0.123
Intestinal flukes ^c^	Abdominopelvic problems due to heavy intestinal fluke infections	0.123
*Opisthorchis* spp.	Abdominopelvic problems due to heavy opisthorchiosis	0.123
*Paragonimus* spp.	Central nervous system problems due to heavy paragonimosis	0.420
	Pulmonary problems due to heavy paragonimosis	0.132
**Organic pollutants**		
Dioxin	Infertility	0.056
	Hypothyroidy due to prenatal exposure	0.019
	Hypothyroidy due postnatal exposure	0.019
**Toxins and allergens**		
Aflatoxin	Hepatocellular carcinoma: diagnosis and primary therapy	0.294
	Hepatocellular carcinoma: metastatic	0.484
	Hepatocellular carcinoma: terminal phase with medication	0.508
	Hepatocellular carcinoma: terminal phase without medication	0.519
Cyanide in cassava	Konzo	0.065
Peanut allergens ^b^	Living with peanut-induced allergy	0.012

^a^ The disability weights for diarrheal disease were defined as a weighted average of the disability weights for mild, moderate and severe diarrhea, with different relative contributions of these severity levels leading to different weighted averages for different diarrheal disease agents.

^b^ Excluded from global burden assessments.

^c^ Includes *Echinostoma* spp., *Fasciolopsis buski*, *Heterophyes* spp., *Metagonimus* spp. and other foodborne intestinal trematode species.

DALYs, and more specifically their YLD component, may be calculated from an incidence or a prevalence perspective. While incidence-based YLDs are defined as the product of the number of incident cases and the duration and disability weight (DW) of the concerned health state, prevalence-based YLDs are defined as the product of the number of prevalent cases and the corresponding DW [13,14]. In the incidence-based approach, all health outcomes, including those in future years, are assigned to the initial event (e.g. exposure to a certain hazard). This approach therefore reflects the future burden of disease resulting from current events. In the prevalence-based approach, on the other hand, the health status of a population is assessed at a specific point in time, and prevalent diseases are attributed to initial events that happened in the past. This approach therefore reflects the current burden of disease resulting from previous events. For burden of FBD studies, the incidence-based YLD approach was deemed the most appropriate approach, because (1) this approach is more sensitive to current epidemiological trends [20]; (2) is more consistent with the hazard-based approach, since it has the point of infection (or primary health effect from exposure) as starting point for the calculations; and (3) is more consistent with the estimation of YLLs, which by definition follows an incidence-based approach, as mortality can be seen as the incidence of death [21]. Nevertheless, the prevalence- and incidence-based approaches yield similar overall results if the epidemiology of disabilities and the population age-structure are constant over time [20]. However, burden estimates for specific age groups will always differ between the prevalence- and incidence-based approaches, because the former assigns the burden to the age at which the burden is experienced, while the latter assigns the burden to the age of disease onset [15].

## CTF Structure and Workflow

**Fig 1:**
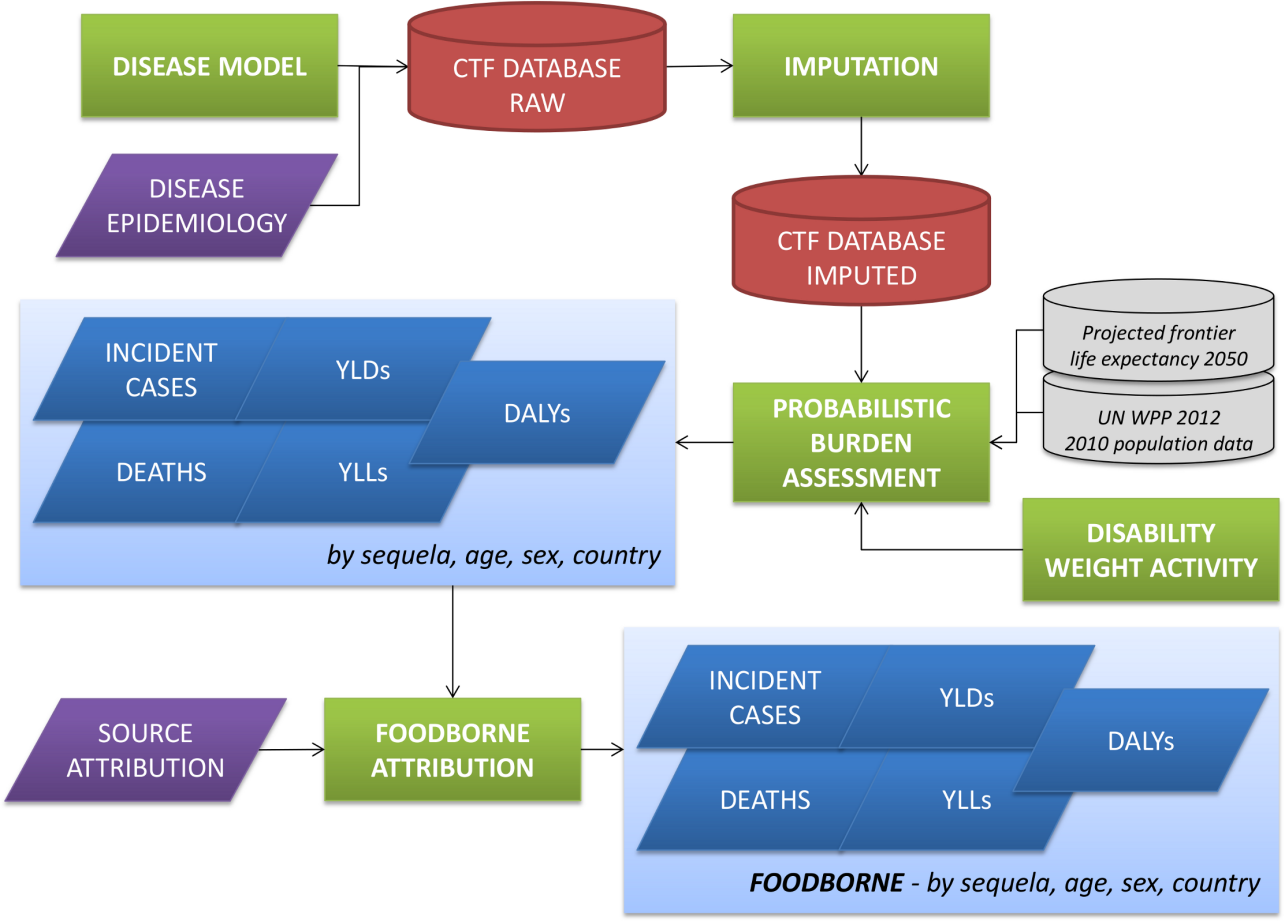
Computational Task Force workflow. CTF = Computational Task Force; YLDs = Years Lived with Disability; YLLs = Years of Life Lost due to mortality; DALYs = Disability-Adjusted Life Years; UN WPP 2012 = United Nations World Population Prospects 2012 Revision.

Fig 1 shows a schematic overview of the CTF workflow. The CTF structure is defined around the six distinctive components of this workflow, i.e., outline of disease models and collection of epidemiological data; design of a database template; development of an imputation model; identification of disability weights; probabilistic burden assessment; and source attribution of the disease burden. All computations were implemented in R version 3.2.0 [22], and the various functions were compiled in the R package 'FERG', which is available as Supporting Information to this manuscript (S1 File) and online at https://github.com/brechtdv/FERG.

### Disease models and epidemiological data

The starting point of the CTF workflow was the outline of disease models for each of the included hazards (as chosen by the hazard-based task forces), and the epidemiological data inputs that parameterized these disease models. To obtain this information, systematic reviews were commissioned and managed by three hazard-based task forces, i.e., the Enteric Diseases Task Force (EDTF), the Parasitic Diseases Task Force (PDTF), and the Chemicals and Toxins Task Force (CTTF). Details are therefore provided elsewhere [23–25].

The course of disease is characterized by various health states (e.g. acute or chronic phases, short-term or long-term sequelae), possibly having different severity levels [8]. A disease model, also referred to as an outcome tree, is a schematic representation of the various health states associated with the concerned hazard, and the possible transitions between these states. A disease model for each hazard was defined by the members and commissioned experts of each hazard-based task force, considering relevant health outcomes supported by evidence identified in the respective reviews. Discussions of the choices and uncertainties in deriving the disease models for specific hazards are provided elsewhere [23–25].

In the context of the CTF, disease models were defined as *computational* disease models, and not merely as *biological* disease models. While biological disease models merely reflect the natural history of disease, computational disease models also reflect the input parameters needed to calculate incidence and mortality of each of the concerned health states. As such, computational disease models are a combination of disease biology and data availability.

Computational disease models may be represented as directed acyclic graphs, defined by parent and child nodes and directed edges (arrows) defining the relationships between nodes. In the CTF framework, parent nodes were either incidence, mortality, YLD or YLL rates, while child nodes were multiplicative elements, such as proportions or ratios (reflecting e.g. the probability of developing a specific symptom following infection, or the proportion of illnesses attributable to the concerned hazard). A specific disease model "language" was developed to denote the relationship and contribution of the different nodes. Rectangles defined parent nodes, and rounded rectangles defined child nodes. Grey nodes did not contribute directly to the DALYs, green nodes contributed YLDs, and red nodes contributed YLLs. Nodes that contributed to the incidence of the index disease were identified by a thick border. S1 Fig gives the disease models for all 36 FERG hazards.

In general, three main approaches can be distinguished for estimating the burden due to a specific hazard in food, i.e., categorical attribution, counterfactual analysis, and risk assessment. S1 Table gives an overview of the modelling strategy applied for each included hazard. As the choice of the modelling strategy was mainly driven by the type of data available, no sensitivity analyses could be performed to triangulate different modelling approaches. Modelling choices were further driven by a strive for consistency with existing WHO Global Health Estimates.

**Fig 2:**
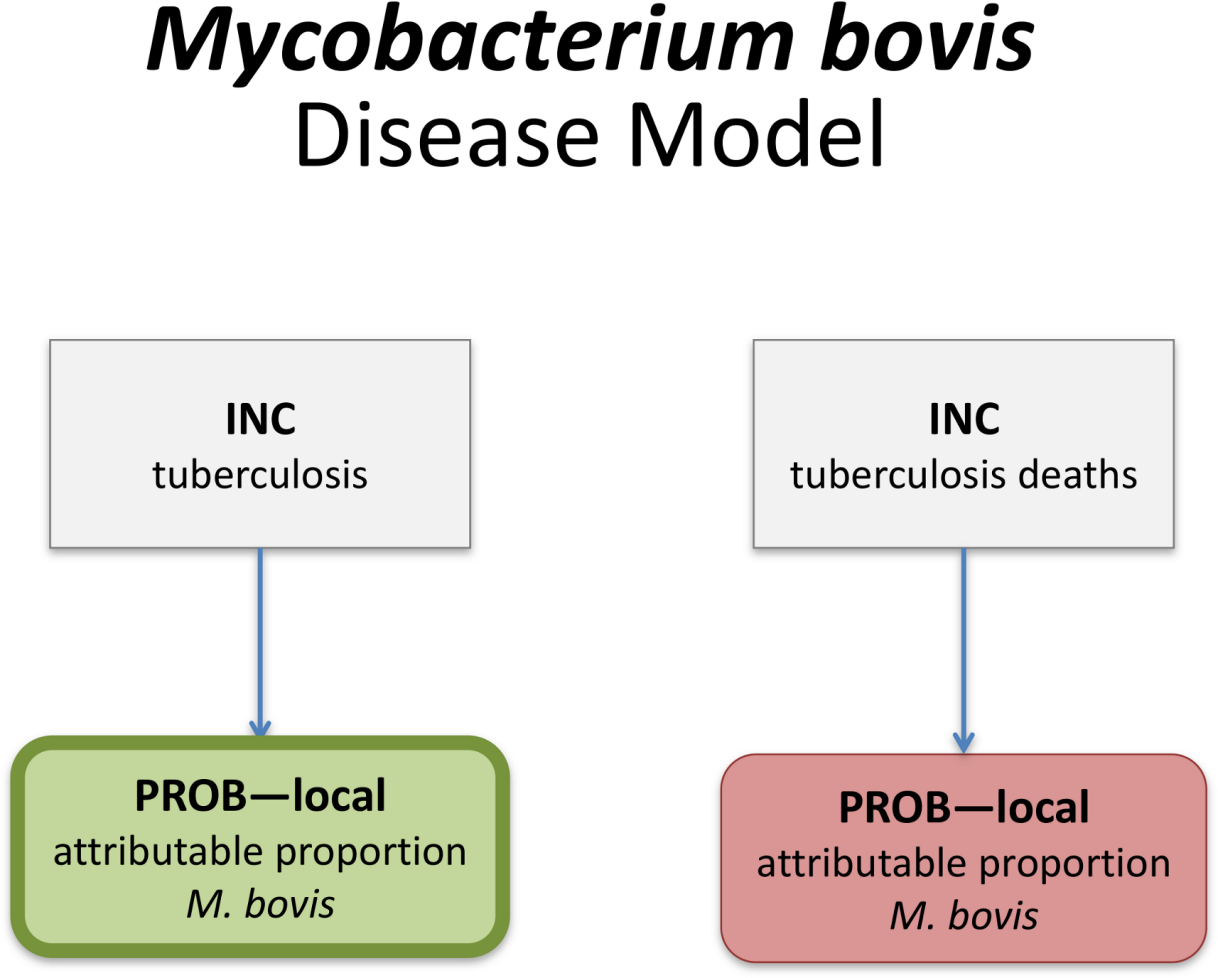
Computational disease model for *Mycobacterium bovis*. Rectangles define parent nodes, while rounded rectangles defined child nodes. Green nodes contribute Years Lived with Disability, and red nodes contribute Years of Life Lost. Nodes contributing to the incidence of the index disease are identified by a thick border. INC = country-specific incidence; PROB–local = country-specific probability.

**Fig 3:**
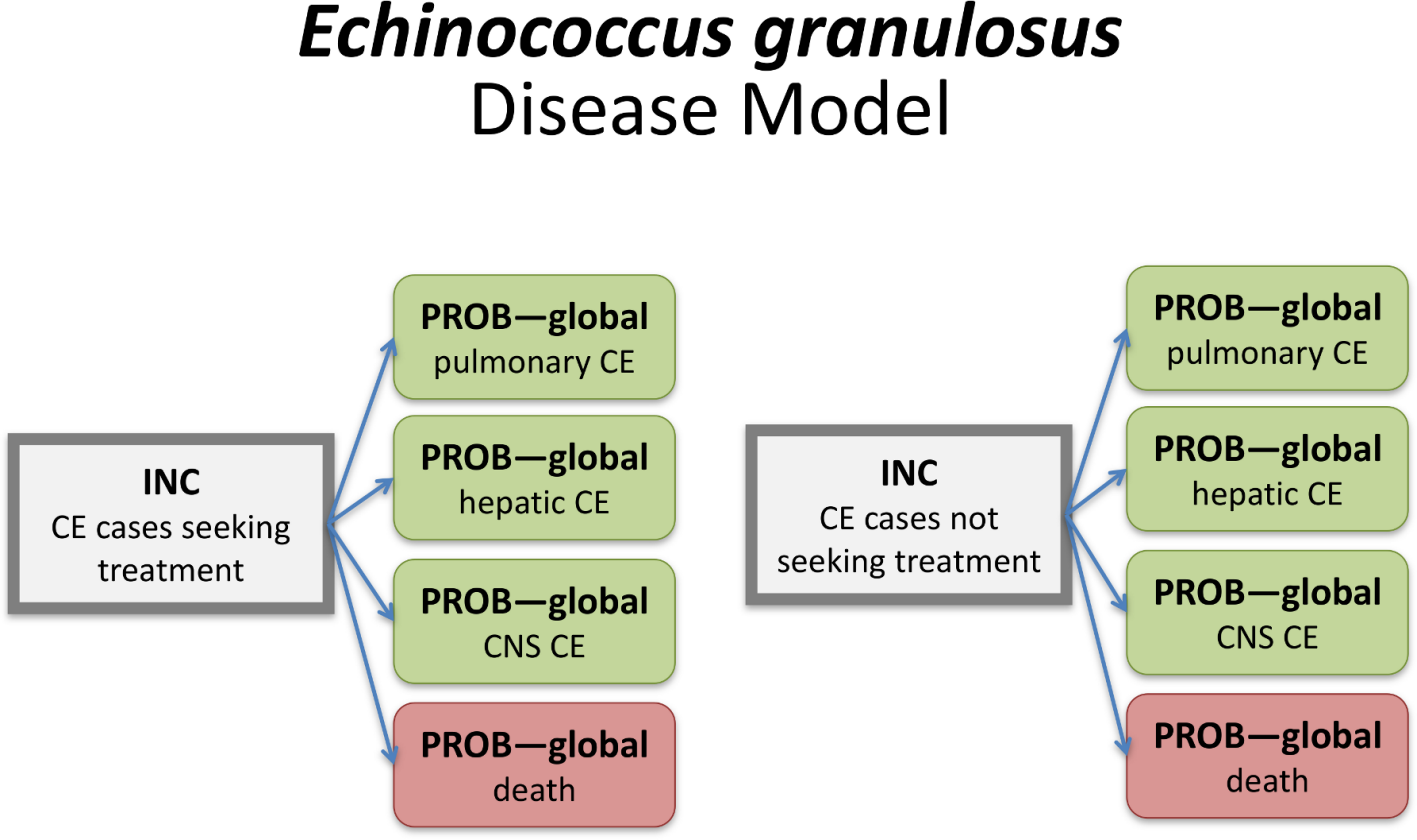
Computational disease model for *Echinococcus granulosus*. Rectangles define parent nodes, while rounded rectangles defined child nodes. Grey nodes do not contribute directly to the DALYs, green nodes contribute YLDs, and red nodes contribute YLLs. Nodes contributing to the incidence of the index disease are identified by a thick border. INC = country-specific incidence; PROB–global = probability applied to all countries; CE = cystic echinococcosis; CNS = central nervous system.

*Categorical attribution* can be used when a foodborne hazard results in an outcome (death or a specific syndrome) that is identifiable as caused by the hazard in individual cases [26]. Following the typology of Devleesschauwer et al. [16], the burden due to a specific hazard can then be calculated using an attributional model (in which the incidence of the symptom is multiplied with the attributable proportion for a given hazard) or a transitional model (in which the incidence of infection with or exposure to the hazard is multiplied with the probability of developing a given symptom). Categorical attribution was applicable for all viral, bacterial and parasitic hazards, and for cyanide in cassava and peanut allergens, and was therefore the standard method used by FERG. Fig 2 shows the computational disease model for *Mycobacterium bovis*, which is characteristic for the attributional models. In this model, the overall incidence and mortality of tuberculosis is multiplied with the proportion attributable to *M*. *bovis*, resulting in the incidence and mortality of *M*. *bovis* tuberculosis. Fig 3 shows the computational disease model for *Echinococcus granulosus*, which is characteristic for the transitional models. In this model, the overall incidence of infection by this parasite was multiplied with child nodes reflecting the probability of developing the concerned health states, resulting in the incidences of the specific health states.

When the hazard elevates the risk of a disease or disability outcome that occurs in the population from other causes as well, causal attribution can only be made statistically, and not on an individual basis. This is the case for many chemicals, including aflatoxin and dioxin. Aflatoxin for instance may increase the risk of hepatocellular carcinoma, but it is not possible to specify that a specific liver cancer case was caused by aflatoxin. In this situation, the standard approach for calculating the burden of environmental exposures is to use a *counterfactual analysis* in which the current disease outcomes with current exposure are compared to the disease outcomes under an alternate exposure (a minimum risk exposure which could be zero, or some accepted background level) [27]. This allows calculation of a population attributable fraction (PAF) that can be applied to the all-cause burden estimates for the relevant disease outcome (the so-called burden envelope), leading to a special case of the attributional model [16]. In the context of FERG, counterfactual analysis was used to estimate the burden of aflatoxin-related hepatocellular carcinoma.

In addition to categorical attribution and counterfactual analysis, which can be considered top-down approaches, FBD burden can also be estimated by a *risk assessment* approach, which can be considered a bottom-up approach. In this approach, the incidences of the specific health states (e.g. impaired male fertility due to prenatal dioxin exposure) are estimated by combining exposure and dose-response data. The dose-response model may for instance define the probability of illness at a given exposure level, which can then be translated into an estimate of the number of incident or prevalent cases expected to occur in the exposed population [25,27]. As this approach does not involve burden attribution, it does not necessarily ensure consistency with existing health statistics. However, risk assessment may be a valid alternative when no burden envelopes exist or when it can be demonstrated that the estimated excess risk is additive to the background risk. In the context of FERG, risk assessment was used to estimate the burden of dioxin-related hypothyroidy and impaired fertility.

### CTF database template

A database template was developed in Excel (Microsoft Corp., Redmond, Washington, USA) to collect the data resulting from the systematic reviews in a standardized way (S2 File). The structure of the database was based on the disease models, with one sheet per node. Three generic sheets were defined: (1) a "RATE" sheet, for rates by country; (2) a "PROB-local" sheet, for proportions or ratios by country; and (3) a "PROB-global" sheet, for a single proportion or ratio that applied to all countries.

Each sheet consisted of four tables for entering (1) the rate or proportion/ratio data; (2) the age distribution; (3) the sex distribution; and (4), if applicable, the duration. Using a drop-down menu, different formats could be selected for entering the input parameters, including a mean and 95% confidence interval; a minimum, most likely and maximum; different percentiles; the shape and rate of a Gamma distribution (for rates); and the shape parameters of a Beta distribution (for proportions). Gamma and Beta distributions were chosen because their domains correspond to those of rates and proportions, respectively, and because their parameters have an intuitive interpretation (i.e., number of cases and sample size or observation time, respectively, number of positives and number of negatives). Likewise, different levels of stratification could be selected for the duration parameters (i.e., none, by age only, by sex only, by age and sex). Age distribution, sex distribution and duration were allowed to vary by country, by defining different "groups" and assigning countries to "groups". Full details on the parameters used for quantifying the burden of the different hazards is available in the appendices to the EDTF, PDTF and CTTF manuscripts [23–25].

### Imputation

Extrapolation or imputation models may be needed when literature searches cannot provide essential epidemiological data such as incidence or mortality rates [28]. These models estimate parameters based on data of neighboring regions or other time periods. The external data used must thus be representative of the selected population, region and time. The CTF developed, tested and evaluated several possible approaches to impute missing incidence data at the country level [29]. This exercise identified several pitfalls in the use of explanatory covariates, such as the potential for overfitting and the arbitrariness in the selection of covariates. Therefore, and further motivated by a strive for parsimony and transparency, we decided to use a log-Normal random effects model as the default model for imputing missing country-level incidence data. We used the subregions as defined in Table 2 as the random effect or cluster variable. This model assumes that the log-transformed incidence rate in country *j* belonging to subregion *i* arises from a Normal distribution with subregion specific mean *μ*_*i*_ and a within-region (= between-country) variance $\sigma_{w}^{2}$. Each subregion specific mean *μ*_*i*_ is in turn assumed to arise from a Normal distribution with mean *μ*_0_ and a between-region variance $\sigma_{b}^{2}$:

$$\text{log}(\theta_{ij})∼Normal(\mu_{i},\sigma_{w}^{2})$$

$$\mu_{i}∼Normal(\mu_{0},\sigma_{b}^{2})$$

**Table 2:** World Health Organization (WHO) Member States by subregion.

Subregion^a^	WHO member states
AFR D	Algeria; Angola; Benin; Burkina Faso; Cameroon; Cape Verde; Chad; Comoros; Equatorial Guinea; Gabon; Gambia; Ghana; Guinea; Guinea-Bissau; Liberia; Madagascar; Mali; Mauritania; Mauritius; Niger; Nigeria; Sao Tome and Principe; Senegal; Seychelles; Sierra Leone; Togo.
AFR E	Botswana; Burundi; Central African Republic; Congo; Côte d'Ivoire; Democratic Republic of the Congo; Eritrea; Ethiopia; Kenya; Lesotho; Malawi; Mozambique; Namibia; Rwanda; South Africa; Swaziland; Uganda; United Republic of Tanzania; Zambia; Zimbabwe.
AMR A	Canada; Cuba; United States of America.
AMR B	Antigua and Barbuda; Argentina; Bahamas; Barbados; Belize; Brazil; Chile; Colombia; Costa Rica; Dominica; Dominican Republic; El Salvador; Grenada; Guyana; Honduras; Jamaica; Mexico; Panama; Paraguay; Saint Kitts and Nevis; Saint Lucia; Saint Vincent and the Grenadines; Suriname; Trinidad and Tobago; Uruguay; Venezuela (Bolivarian Republic of).
AMR D	Bolivia (Plurinational State of); Ecuador; Guatemala; Haiti; Nicaragua; Peru.
EMR B	Bahrain; Iran (Islamic Republic of); Jordan; Kuwait; Lebanon; Libyan Arab Jamahiriya; Oman; Qatar; Saudi Arabia; Syrian Arab Republic; Tunisia; United Arab Emirates.
EMR D	Afghanistan; Djibouti; Egypt; Iraq; Morocco; Pakistan; Somalia; South Sudan^b^; Sudan; Yemen.
EUR A	Andorra; Austria; Belgium; Croatia; Cyprus; Czech Republic; Denmark; Finland; France; Germany; Greece; Iceland; Ireland; Israel; Italy; Luxembourg; Malta; Monaco; Netherlands; Norway; Portugal; San Marino; Slovenia; Spain; Sweden; Switzerland; United Kingdom.
EUR B	Albania; Armenia; Azerbaijan; Bosnia and Herzegovina; Bulgaria; Georgia; Kyrgyzstan; Montenegro; Poland; Romania; Serbia; Slovakia; Tajikistan; The Former Yugoslav Republic of Macedonia; Turkey; Turkmenistan; Uzbekistan.
EUR C	Belarus; Estonia; Hungary; Kazakhstan; Latvia; Lithuania; Republic of Moldova; Russian Federation; Ukraine.
SEAR B	Indonesia; Sri Lanka; Thailand.
SEAR D	Bangladesh; Bhutan; Democratic People's Republic of Korea; India; Maldives; Myanmar; Nepal; Timor-Leste.
WPR A	Australia; Brunei Darussalam; Japan; New Zealand; Singapore.
WPR B	Cambodia; China; Cook Islands; Fiji; Kiribati; Lao People's Democratic Republic; Malaysia; Marshall Islands; Micronesia (Federated States of); Mongolia; Nauru; Niue; Palau; Papua New Guinea; Philippines; Republic of Korea; Samoa; Solomon Islands; Tonga; Tuvalu; Vanuatu; Viet Nam.

^a^ The subregions are defined on the basis of child and adult mortality as described by Ezzati et al. [49]. Stratum A: very low child and adult mortality, Stratum B: low child mortality and very low adult mortality, Stratum C: low child mortality and high adult mortality, Stratum D: high child and adult mortality, and Stratum E: high child mortality and very high adult mortality. The use of the term “subregion” here and throughout the text does not identify an official grouping of WHO Member States, and the “subregions” are not related to the six official regions. AFR = African Region; AMR = Region of the Americas; EMR = Eastern Mediterranean Region; EUR = European Region; SEAR = South-East Asia Region; WPR = Western Pacific Region.

^b^ South Sudan was reassigned to the African Region in May 2013. As this study relates to time periods prior to this date, estimates for South Sudan were included in the Eastern Mediterranean Region.

After fitting this hierarchical random effects model to the available data, incidence values for countries with no data were imputed based on the resulting posterior predictive distributions. In other words, we represented missing incidence data by log-Normal distributions based on the fitted mean and variance parameters. For countries in a subregion where none of the countries had data, the log-incidence was imputed as multiple random draws from a Normal distribution with mean equal to the fitted global intercept *μ*_0_ and variance equal to the sum of the fitted between-region variance $\sigma_{b}^{2}$ and the fitted within-region variance $\sigma_{w}^{2}$ (thus imputing the log-incidence as that of a “random” country within a “random” subregion, with the uncertainty interval describing the variability between and within subregions):

$$\text{log}(\theta_{ij}^{*})∼Normal(\mu_{0},\sigma_{b}^{2}+\sigma_{w}^{2})$$

For countries in a subregion where at least one of the other countries had data, the log-incidence was imputed as multiple random draws from a Normal distribution with mean equal to the fitted region-specific intercept *μ*_*i*_ and variance equal to the fitted within-region variance $\sigma_{w}^{2}$ (thus imputing the log-incidence as that of a “random” country within the concerned subregion, with the uncertainty interval describing the variability within subregions):

$$\text{log}(\theta_{ij}^{*})∼Normal(\mu_{i},\sigma_{w}^{2})$$

When countries were considered free from exposure through the food chain, they were excluded from the imputation model and thus did not contribute to the subregional estimates. This was the case for *Brucella* spp., as discussed in [23], and *Echinococcus granulosus*, as discussed in [24]. For countries with available incidence data, no imputation was performed. The incidence data used in the probabilistic burden assessments were thus a combination of actual data and imputed estimates. No additional step was included to correct incidence data for potential underreporting, as this was already captured by the previous steps of the framework. Indeed, for the hazards that used an attributional model, disease envelopes were used that had already been corrected for underreporting, while for other hazards we directly drew on GBD 2010 estimates (S1 Table). For the remaining hazards, either epidemiological data were used that did not need (further) correction, or the underreporting factor was included in the disease model (which was the case for *Trichinella* spp. and cyanide in cassava).

For aflatoxin, the same random effects model was used to extrapolate PAFs, but now using logit-transformed instead of log-transformed values.

The model was implemented in a Bayesian framework, using independent Normal(0, 1e5) priors for *μ*_0_ and all *μ*_*i*_; a Uniform(0, 10) prior for σ_*w*_; and a Folded-t(1) prior for σ_*b*_, as suggested by Gelman [30]. Sensitivity analyses using Gamma priors for the variance parameters did not yield meaningful differences. The model was run in JAGS [31] through the 'rjags' package in R [32]. After a burn-in of 5000 iterations, another 5000 iterations were retained for inference. Two chains were run, and convergence was ascertained through density and trace plots, and the multivariate potential scale reduction factor (or Brooks-Gelman-Rubin diagnostic). The applied JAGS code is given in S1 Code.

A crucial assumption made by this imputation model is that missing data were considered "missing at random" (MAR), meaning that missingness was independent of the unobserved data, given the observed data [33,34]. In our case, this assumption implied that, within each subregion, countries with data provided unbiased information on those without data, and that, across subregions, subregions with data provided unbiased information on those without data. For five hazards (*Bacillus cereus*, *Clostridium perfringens*, *Clostridium botulinum*, *Staphylococcus aureus*, and peanut allergens), however, only data from high-income subregions, i.e., subregions A or B, could be retrieved. In those instances, the assumption of MAR was clearly violated, and it was decided not to extrapolate those data to the rest of the world. As a result, those hazards were excluded from the global burden of disease estimates [35].

S1 Table shows which imputation strategy was used for each of the included hazards. For the four intoxications, peanut allergens, and cyanide in cassava, the default random effects model was not used because of the limited number of data points. Instead, the burden for each concerned country was imputed as draws from a Uniform distribution defined by the lowest and highest globally observed incidence or mortality rates. To ensure consistency with results of the Child Health Epidemiology Reference Group (CHERG), alternative imputation approaches were applied for estimating etiological fractions for the eleven diarrheal agents [23,36,37]. For six other hazards, no imputation had to be performed because data were used that had already been imputed. This was the case for hepatitis A virus, *Salmonella* Typhi, *Salmonella* Paratyphi, and *Taenia solium*, for which GBD 2010 data were used, and for *Mycobacterium bovis* and *Trichinella* spp., for which other published data were used [38,39].

### Disability weights

DALYs incorporate the severity of health states through the DW, reflecting the corresponding relative reduction in health on a scale from zero to one. DWs for several health states have been derived for the GBD studies and for various national burden of disease studies [40]. To ensure comparability, the CTF adopted the DWs that were used for WHO's Global Health Estimates [15]. These DWs were based on those derived for the GBD 2010 study [41], but with an alternative value for primary infertility (i.e., 0.056 instead of 0.011). The latter revision was motivated by an analysis showing that the GBD 2010 weights undervalued the health states associated with infertility [15]. For dioxin-induced hypothyroidy, we adopted the GBD 2013 DW for hypothyroidy, as this health state was not included in the GBD 2010 DW study [42].

Several FBDs present with unique clinical signs for which no DWs have been derived. Acute trichinellosis, for instance, typically presents with myalgia and facial edema, for which no specific DWs are available [39]. When DWs were missing, proxy health states were selected by a medical expert and DW expert in the CTF and confirmed by disease experts in the hazard specific task forces. In other instances, DWs were available for severity levels that were not explicitly considered in the disease models. For diarrhea, for instance, DWs were available for mild, moderate and severe diarrhea, although the disease models only included diarrhea as such. In those cases, weighted averages were calculated based on published reviews of severity distributions, avoiding an over- or underestimation of YLDs that would occur if only one DW would have been selected. Table 1 lists the DWs used for the different FERG health states; S2 Table provides further details on their derivation.

### Probabilistic burden assessment

For each hazard, incidence, mortality, YLD, YLL and DALY rates were calculated for 11 age groups (<1; 1–4; 5–14; 15–24; 25–34; 35–44; 45–54; 55–64; 65–74; 75–84; ≥85) and both sexes. When necessary, age and sex specific rates were obtained by multiplying the overall rates with outcome specific age and sex distributions. The reference year for the calculation of absolute numbers was 2010, with population estimates obtained from the 2012 revision of the United Nations World Population Prospects [43]. All estimates were generated per country, and subsequently aggregated per subregion, per region, and globally (Table 2). The resulting estimates are presented in three hazard-specific papers [23–25] and in a summary paper [35]. The results may also be accessed and explored through an online tool (https://extranet.who.int/sree/Reports?op=vs&path=/WHO_HQ_Reports/G36/PROD/EXT/FoodborneDiseaseBurden), which shows the breadth and flexibility of our framework in terms of aggregating and reporting estimates.

The duration component of the YLDs is defined as the average observed duration until the next health state (e.g., remission or death). For calculating YLDs when duration was lifelong, we therefore used the country-specific life expectancy (LE) [43] as duration. The YLLs, on the other hand, are essentially calculated as the number of cause-specific deaths multiplied by a loss function specifying the years lost for deaths as a function of the age at which death occurs [15]. In standard DALY methodology, the time component of the YLLs is defined as the ideal residual life expectancy a person would have if the world would be free from disease and provide maximal access to health care. In accordance with the WHO Global Health Estimates, we used the highest projected LE for 2050 as normative LE for calculating YLLs [15]. This LE table has a LE at birth of 92, higher than that of the LE tables used in the GBD studies, which were based on current death rates [13,14]. Since even for the lowest observed death rates there are a proportion of deaths which are preventable or avertable, the highest projected LE for the year 2050 was deemed to better represent the maximum life span of an individual in good health, while acknowledging that it may still not represent the ultimate achievable human life span [15]. In line with current global burden of disease assessments, no age weighting or time discounting was applied [14,15]. HIV infected invasive salmonellosis cases and deaths, and HIV infected *M*. *bovis* deaths, were excluded from the burden estimates. No further corrections were made for possible co-morbidities.

Parameter uncertainty was taken into account by performing the burden assessments in a probabilistic framework. Ten thousand Monte Carlo (parametric bootstrap) simulations of the input parameters were generated to calculate 10,000 estimates of incidence, mortality, YLD, YLL and DALY rates. These 10,000 estimates were then summarized by their median and a 95% uncertainty interval defined as the 2.5^th^ and 97.5^th^ percentile of the distribution of estimates. Special care was taken to deal with correlated uncertainties, for instance when the disease model included "global" probabilities (e.g. when it was assumed that the probability of developing a certain health state following infection was the same for each country). In such cases, a vector of random probabilities was simulated only once and applied to the different countries, instead of incorrectly simulating a new, independent vector of random probabilities for each country.

### Source attribution

The main aim of FERG was to quantify the disease burden resulting from foodborne exposure to potentially foodborne hazards. However, many of the hazards considered are not transmitted solely by food, but have several potential exposure routes (e.g. direct contact with animals, human-to-human transmission, and waterborne transmission). For certain hazards, it was therefore necessary to attribute a proportion of their overall burden to foodborne exposure (S1 Table).

Some hazards were considered 100% foodborne, i.e., *Listeria monocytogenes*, *M*. *bovis*, all foodborne trematodes, *T*. *solium*, *Trichinella* spp., aflatoxin, cyanide in cassava, dioxin and peanut allergens.

For the remaining hazards, a structured expert elicitation using Cooke's Classical Method was conducted to attribute burden to different exposure routes, providing hazards-specific estimates for each exposure route per subregion [44]. This process yielded a probabilistic estimate of the proportion foodborne, in the form of an empirical cumulative density function from which random samples could be drawn. Foodborne cases, deaths, YLDs, YLLs and DALYs were then obtained by multiplying the vectors of random values for these parameters with a vector of random values for the proportion foodborne. As before, the perfect correlation of uncertainty was dealt with by simulating only one vector of random foodborne proportions per subregion, and by applying this vector to all parameters of all countries within the concerned subregion.

## Traceability and Transparency

The process described above was conducted in an iterative way, in which FERG members and disease experts were given the opportunity to review and comment on preliminary results. Per iteration, a time-stamped PDF report was generated containing the most relevant outputs, and shared through an online file hosting service. When needed, teleconferences were organized to resolve outstanding issues. A dedicated teleconference was organized to explain the imputation model, its assumptions and limitations. In June 2014, a face-to-face meeting was held in which the methods were reviewed and preliminary results were discussed with all FERG task force members.

## Discussion

This paper reviewed the methodological framework developed to generate the first systematic quantification of the global burden of FBDs. Details are provided on the underlying methods and assumptions, and tools have been made available for the reader to explore and use. Burden of foodborne disease estimates from these calculations are provided in the papers from the hazard-based task forces.

Although our framework is the first framework for quantifying the global burden of FBDs, other methodological frameworks for estimating global burden have been developed, most notably the Global Burden of Cancer study [45] and the Global Burden of Disease study 2010 and 2013 [14,46]. Compared with these efforts, we present a framework that is centered on the quantification of input parameter uncertainty, and in which modelling plays a minimal role. By retaining the link between the input data (and their uncertainties) and the outputs, our framework provides the opportunity to identify regions and hazards with the most uncertain data, highlighting areas for further research in order to produce more accurate and precise burden data. As such, the FERG philosophy builds on that of the Dutch burden of foodborne disease studies [18] and the Burden of Communicable Diseases in Europe (BCoDE) study [8,19], but expands the scope by being more comprehensive in terms of hazards and geographical coverage, and by adding data imputation and source attribution to the framework.

To overcome the inevitable problem of missing data, we imputed missing data using a hierarchical random effects model as a default. Although the use of explanatory covariates such as eating habits or income levels is often considered in these exercises, we decided not to pursue such models driven by our earlier model evaluations and comparisons [29]. The choice of our default imputation model was further motivated by a strive for parsimony and transparency, while recognizing that other approaches could be used for stand-alone studies. Our imputation model furthermore assumed that missing data were MAR, i.e., that, across subregions and within each specific subregion, disease incidence and missingness were not associated [33,34]. This is a strong assumption, and led to the exclusion of five hazards for which the assumption was clearly violated, i.e., *Bacillus cereus*, *Clostridium perfringens*, *Clostridium botulinum*, *Staphylococcus aureus*, and peanut allergens. For the remaining hazards, it is difficult to evaluate the validity of this assumption, as this would require a comparison of incidence data in countries with data versus countries without data, which per definition is not possible [29]. As a result, this assumption is made in all global burden of disease studies, even though this is not always explicitly mentioned.

**Fig 4:**
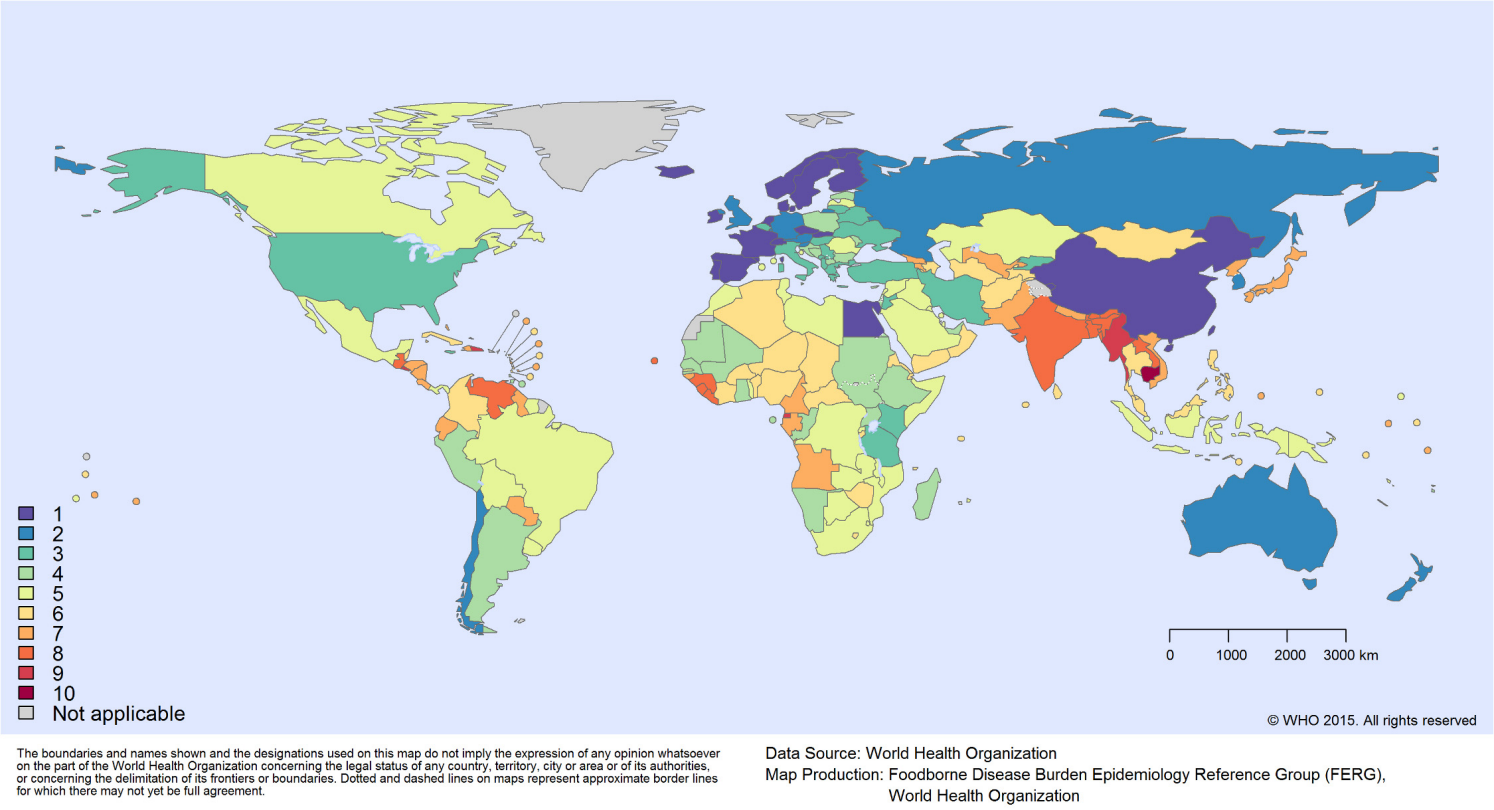
Number of imputed hazards by WHO member state.

For countries in subregions without any data, our model resulted in relatively large uncertainty intervals, as these took into account both variability between and within subregions. Of the 14 hazards that used the random effects imputation model, SEAR B was the subregion for which most often no data could be identified (i.e., for 4 hazards), followed by AMR A, AMR D and SEAR D (for 2 hazards each) (S3 Table). At a country-level, Cambodia was the country with the highest number of data gaps (i.e., for 10 hazards) (Fig 4).

A major limitation of the current framework is that it does not provide for modelling time trends in disease burden. However, the main objective of FERG was to estimate, for the first time, the global burden of FBDs. This sets a baseline against which future trends can be evaluated.

We excluded HIV infected invasive salmonellosis cases and deaths, and HIV infected *M*. *bovis* deaths, from the burden estimates. We did not correct for other co-morbidities, although some foodborne hazards, such as *Listeria*, are often linked with co-morbidities such as cancer or diabetes [47]. Some authors have corrected DALYs for co-morbidities by applying a reduced LE [18]. In the GBD 2010 and GBD 2013 studies, which used a prevalence perspective for estimating YLDs, comorbidities were taken into account by assuming conditional independence between disease prevalences and a multiplicative model for combining DWs [48]. Similar methods have not yet been described for incidence-based YLDs.

No DWs were available for some FBD outcomes; these had to be mapped to proxy health states for which DWs were available. When DWs were available for distinct severity levels, over- or underestimation of the burden was avoided by using weighted averages based on published reviews of severity distributions.

By quantifying the burden of 36 foodborne hazards in a single framework, we generated comparable estimates and avoided overestimation which is typical for single-cause burden studies [3]. For diarrheal hazards, we avoided overestimation by using multi-cause studies to derive etiological fractions and by applying an established diarrhea envelope [36]. Attributional models were also used for tuberculosis due to *M*. *bovis*, epilepsy due to *T*. *solium* and hepatocellular carcinoma due to aflatoxin, ensuring consistency with the respective burden envelopes. For the burden due to *Ascaris* spp., hepatitis A virus, *Salmonella* Typhi, and *Salmonella* Paratyphi, we directly drew on GBD 2010 estimates. For the remaining hazards, however, in absence of well-accepted envelopes or sufficient scientific evidence on etiological fractions, attributional models could not be applied. Instead, either a transitional model or a risk assessment approach was applied, using the best possible data available (S1 Table). Inevitably, however, this might have led to over- or underestimations compared to attributional models.

## Conclusion

We developed a unique methodological framework for estimating the global burden of FBDs, in which methodological simplicity and transparency are key elements. It would be recommended to transform the current tools into a user-friendly national toolkit for estimating and monitoring food safety at the local level. Such a toolkit could incorporate FERG estimates such as source attribution, so that countries lacking such data can still estimate the burden of foodborne diseases.

## Supporting Information

S1 Code JAGS code for Bayesian log-Normal Random Effects Model. (DOC)

S1 Fig Disease models for all FERG hazards. (PDF)

S1 File FERG: DALY Calculation Framework for WHO/FERG. R package version 0.1.0.
(GZ) Click here for additional data file.

S2 File FERG database template. (XLSX)

S1 Table Modelling strategies for the hazards included in the WHO global burden of foodborne disease estimates. (PDF)

S2 Table FERG hazards, causally related health states and corresponding disability weights (DWs). The fourth column describes how the various DWs were derived. The source for the DWs is the Global Burden of Disease 2010 Study, unless stated otherwise.
(PDF)

S3 Table Availability of data for estimating the global and regional burden of foodborne disease. (XLSX)
