# Supplementary material for: Methodological Framework for World Health Organization Estimates of the Global Burden of Foodborne Disease
Source: PLoS One. 2015 Dec 3;10(12):e0142498. doi: 10.1371/journal.pone.0142498 (PMC4668830; doi:10.1371/journal.pone.0142498)
Supplement: S1 Code — (DOC) [file pone.0142498.s001.doc]

**Code S1**

*Bayesian log-Normal Random Effects Model: JAGS code*

model {

## Likelihood, specified using nested indexing.

## N = no. countries, each of which belongs to 1 of NR regions

for (i in 1:N) {

log_y[i] ~ dnorm(mu[reg[i]], tau)

}

## Intercept can differ across regions

for (r in 1:NR) {

mu[r] ~ dnorm(mu.c, mu.tau)

}

## Priors

mu.c ~ dnorm(0, 0.00001)

## within-region sd ~ Unif(0,10)

tau <- 1 / pow(sd, 2)

sd ~ dunif(0, 10)

## between-region sd ~ Folded t(1)

mu.tau <- 1 / pow(sd.tau, 2)

sd.tau <- abs(z) / sqrt(gamma)

z ~ dnorm(0, inv.B.squared)

inv.B.squared <- 1 / pow(1, 2)

gamma ~ dgamma(0.5, 0.5)

}
