# Supplementary material for: Methodological Framework for World Health Organization Estimates of the Global Burden of Foodborne Disease
Source: PLoS One. 2015 Dec 3;10(12):e0142498. doi: 10.1371/journal.pone.0142498 (PMC4668830; doi:10.1371/journal.pone.0142498)
Supplement: S1 Fig — (PDF) [file pone.0142498.s002.pdf]

Norovirus
Disease Model

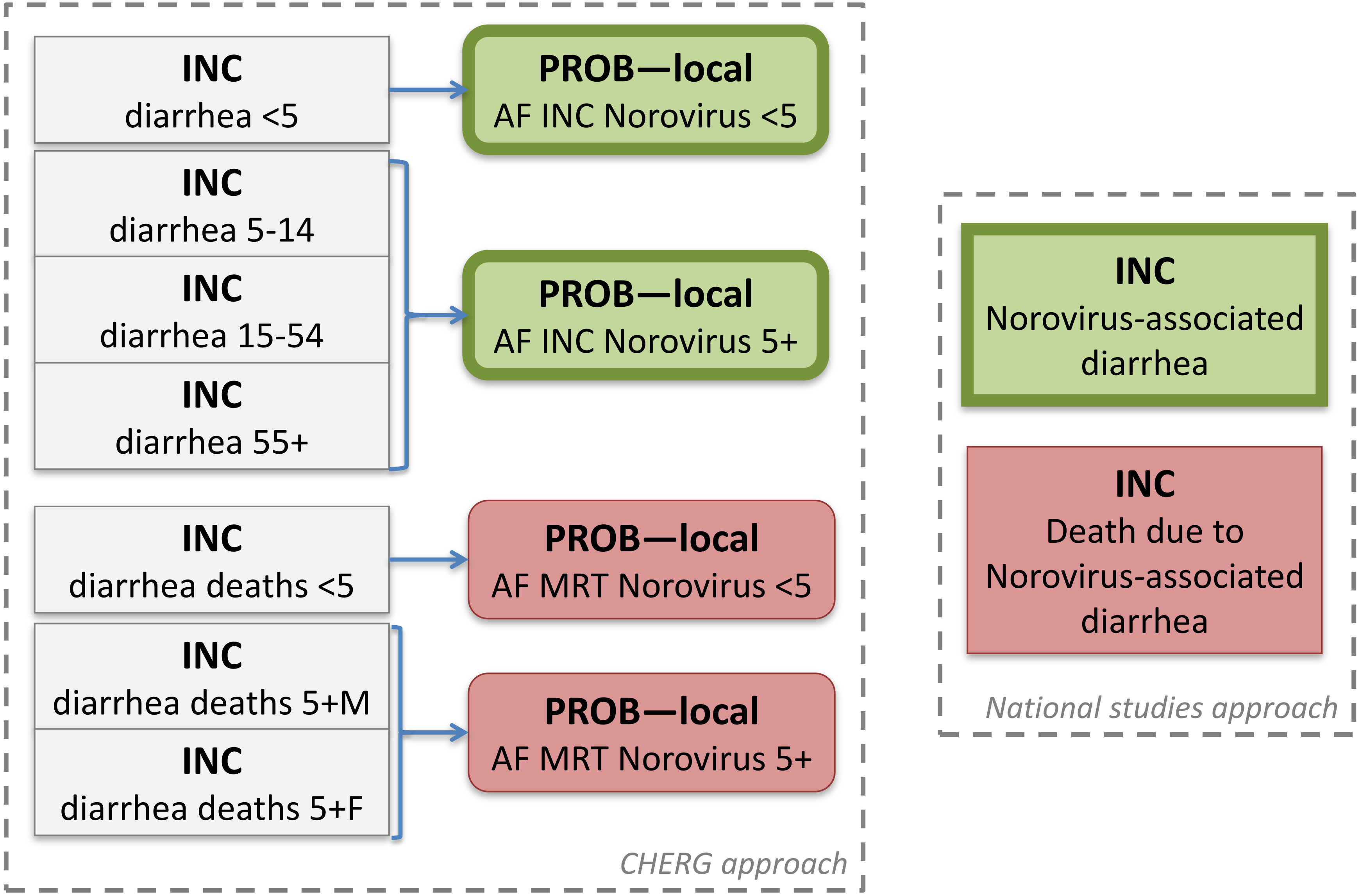

Campylobacter spp.
Disease Model

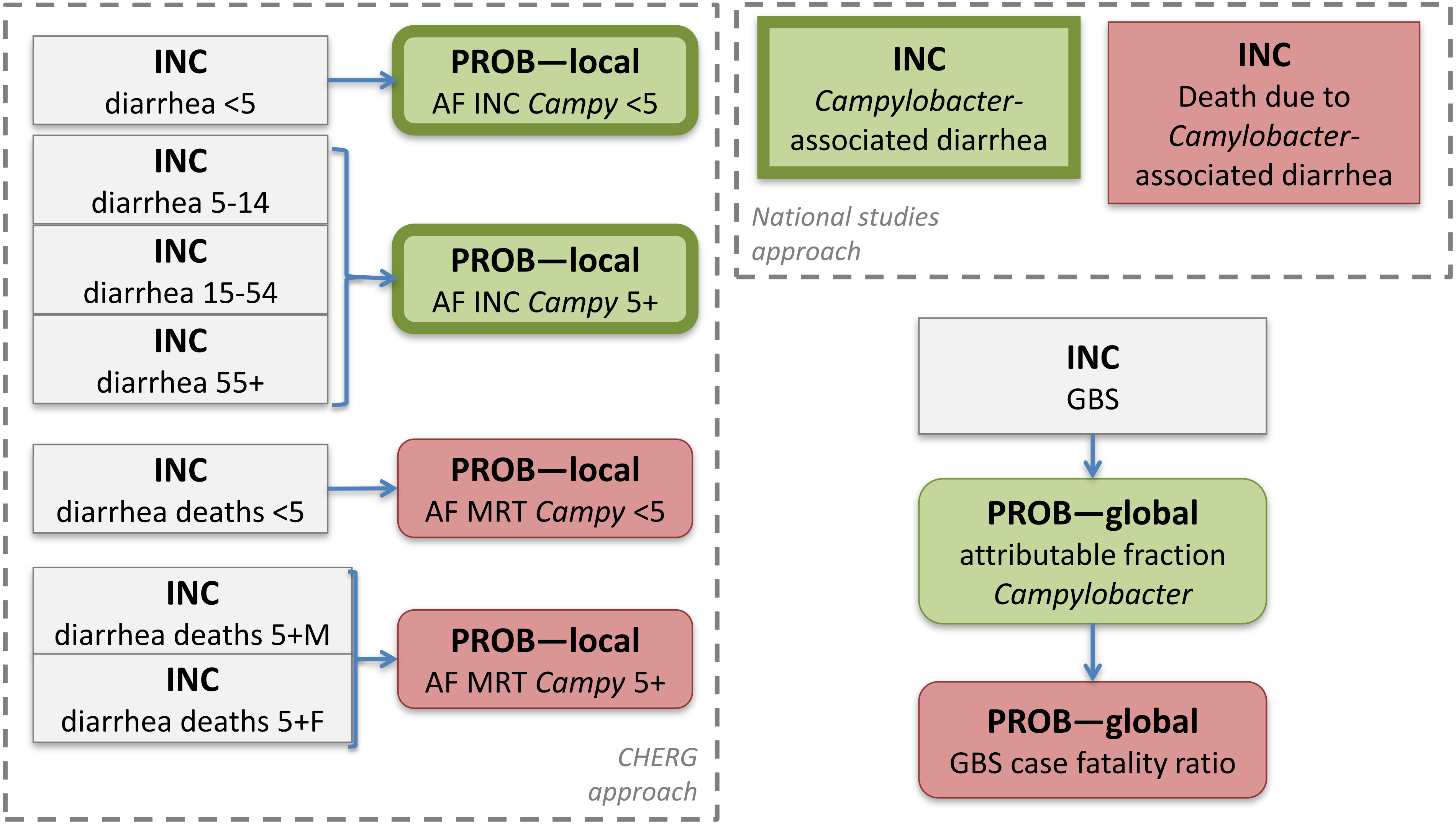

EPEC
Disease Model

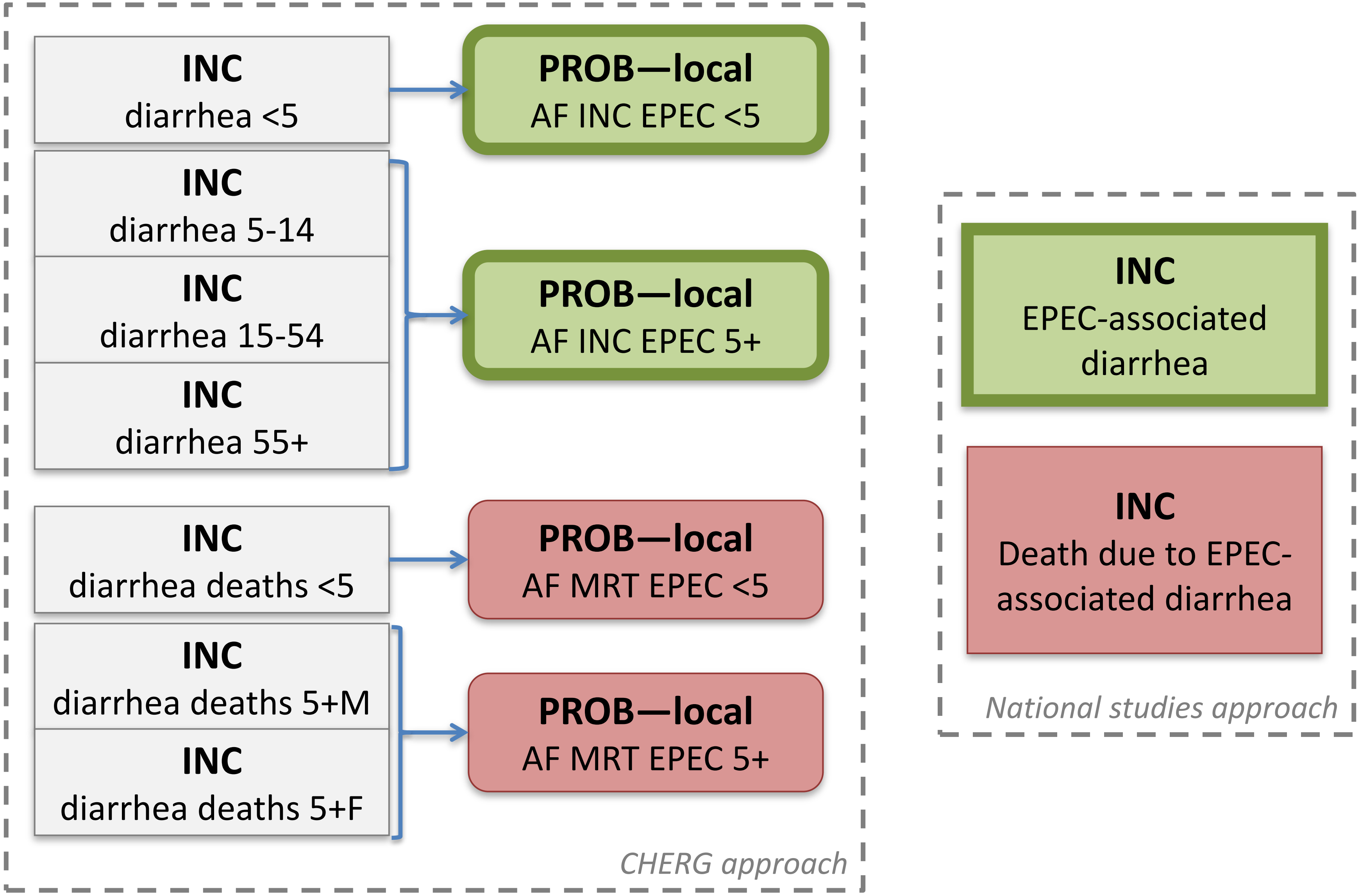

ETEC
Disease Model

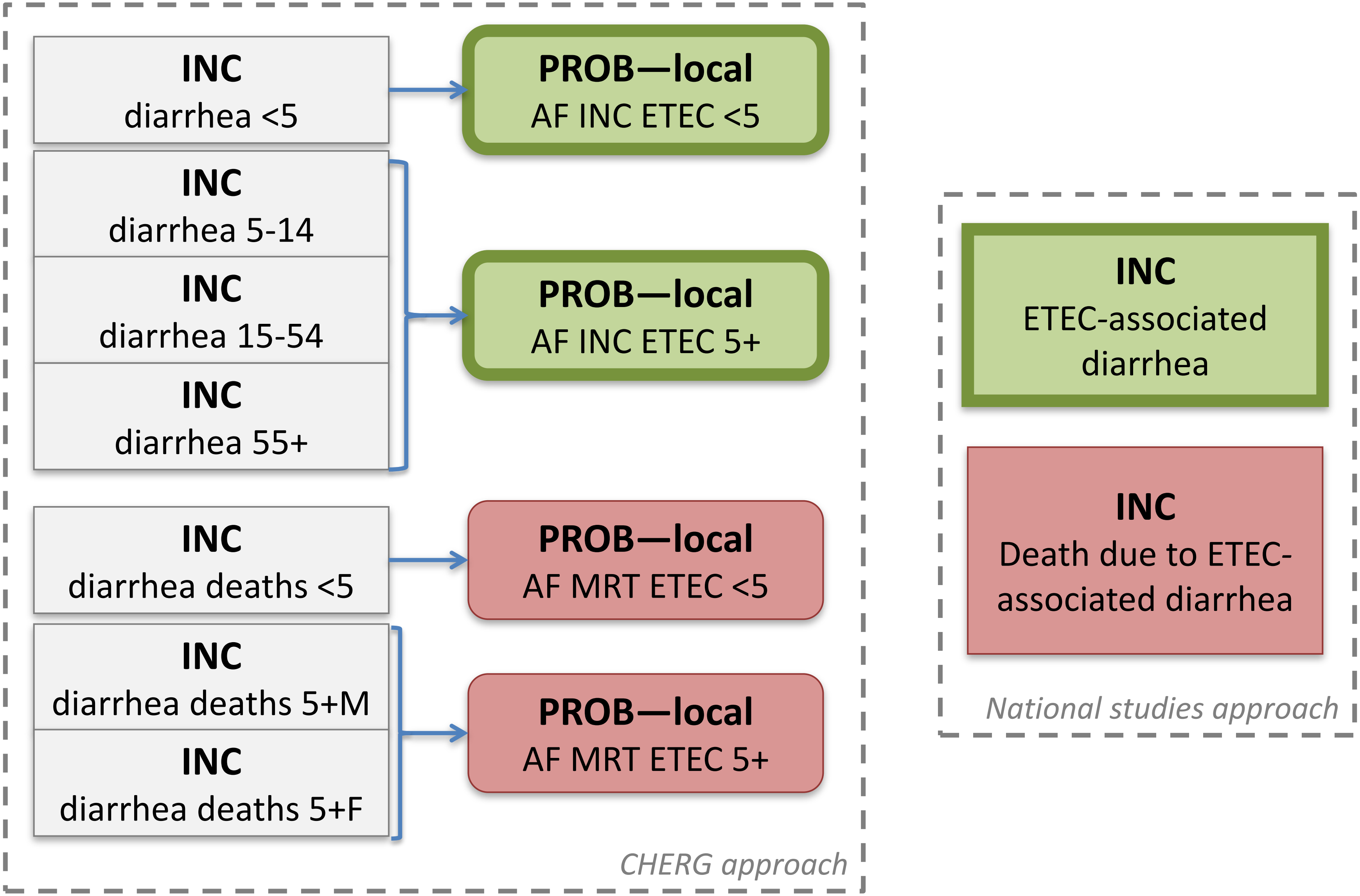

STEC
Disease Model

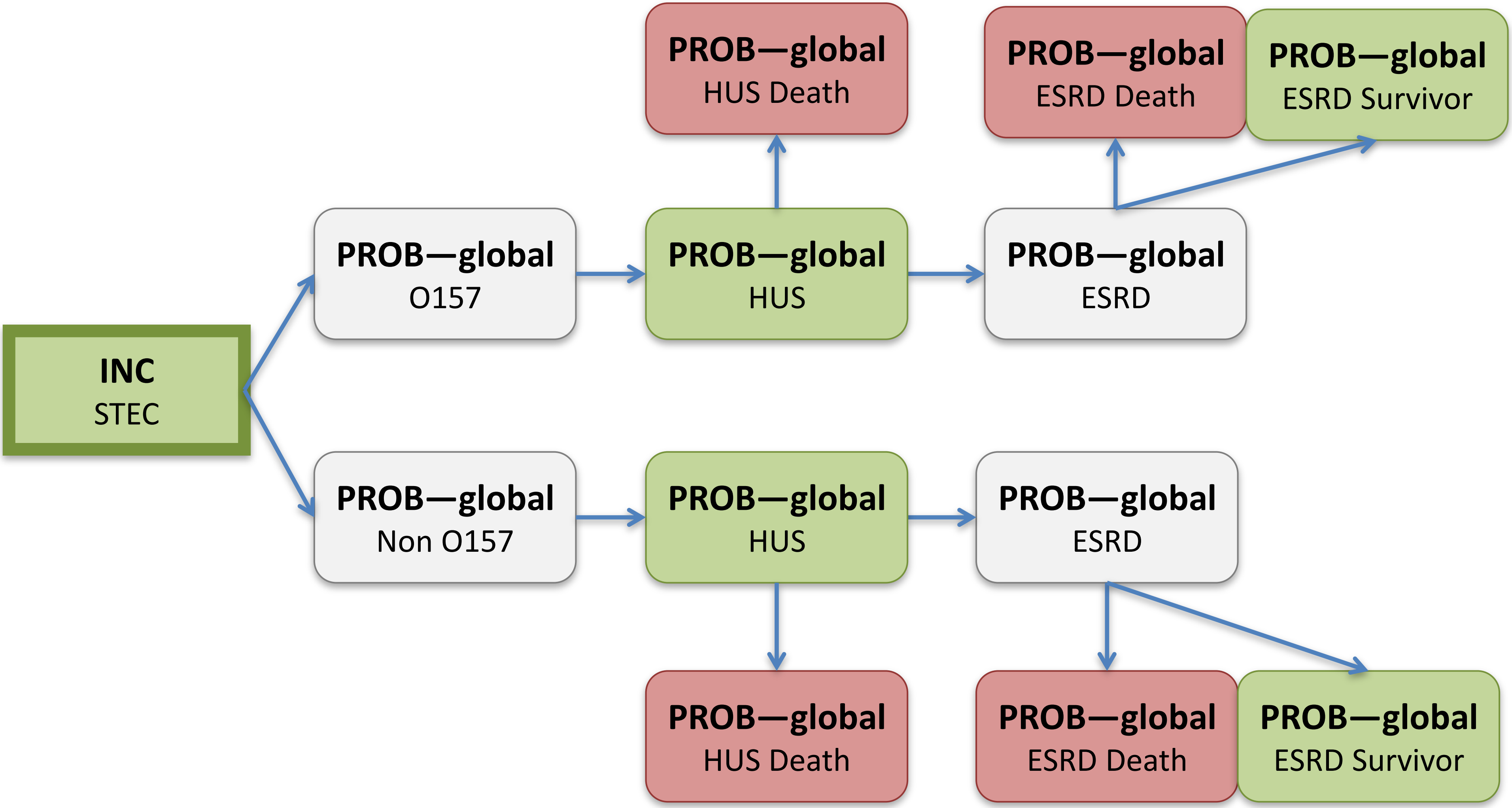

Salmonella enterica
Disease Model

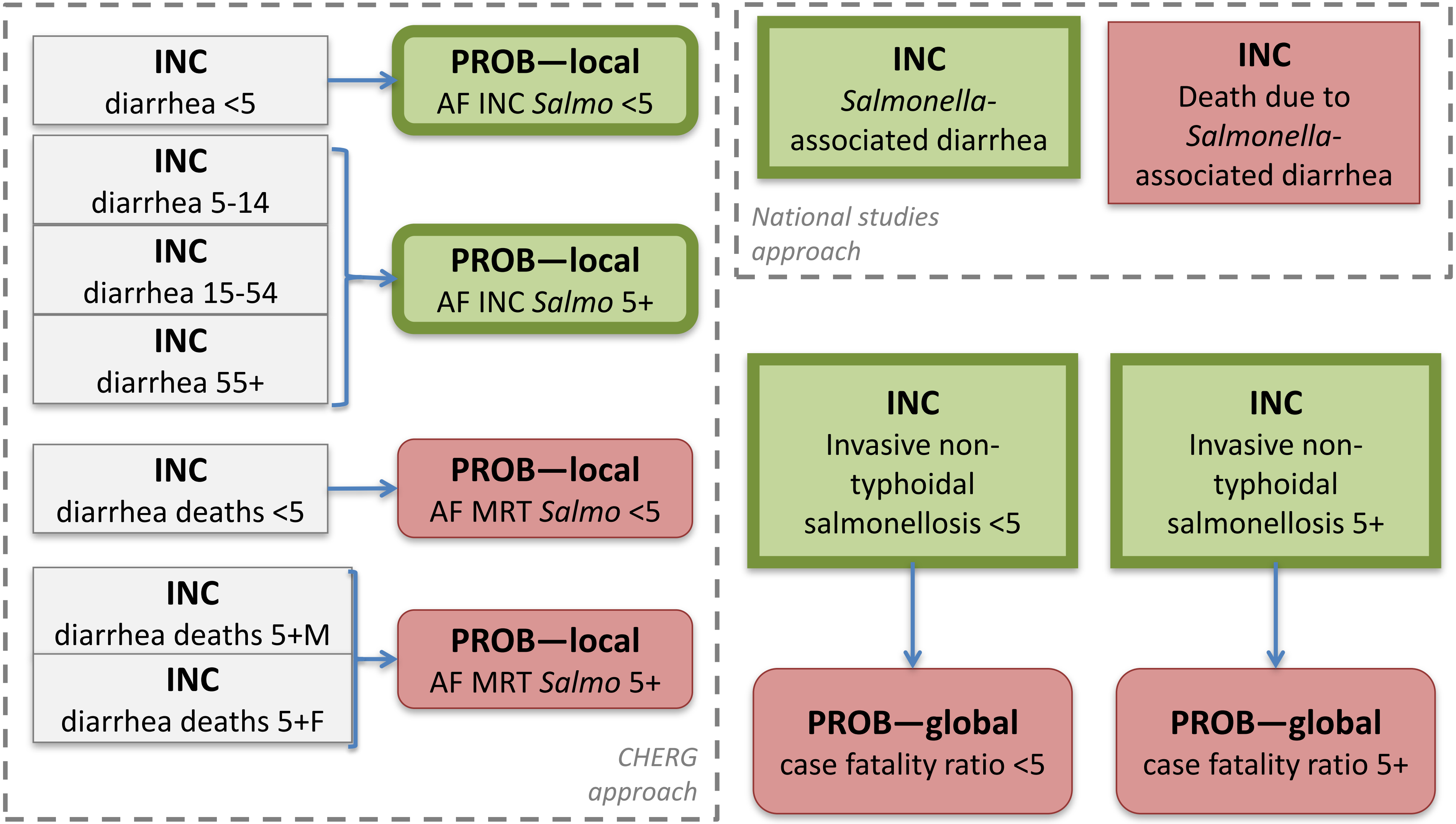

Shigella spp.
Disease Model

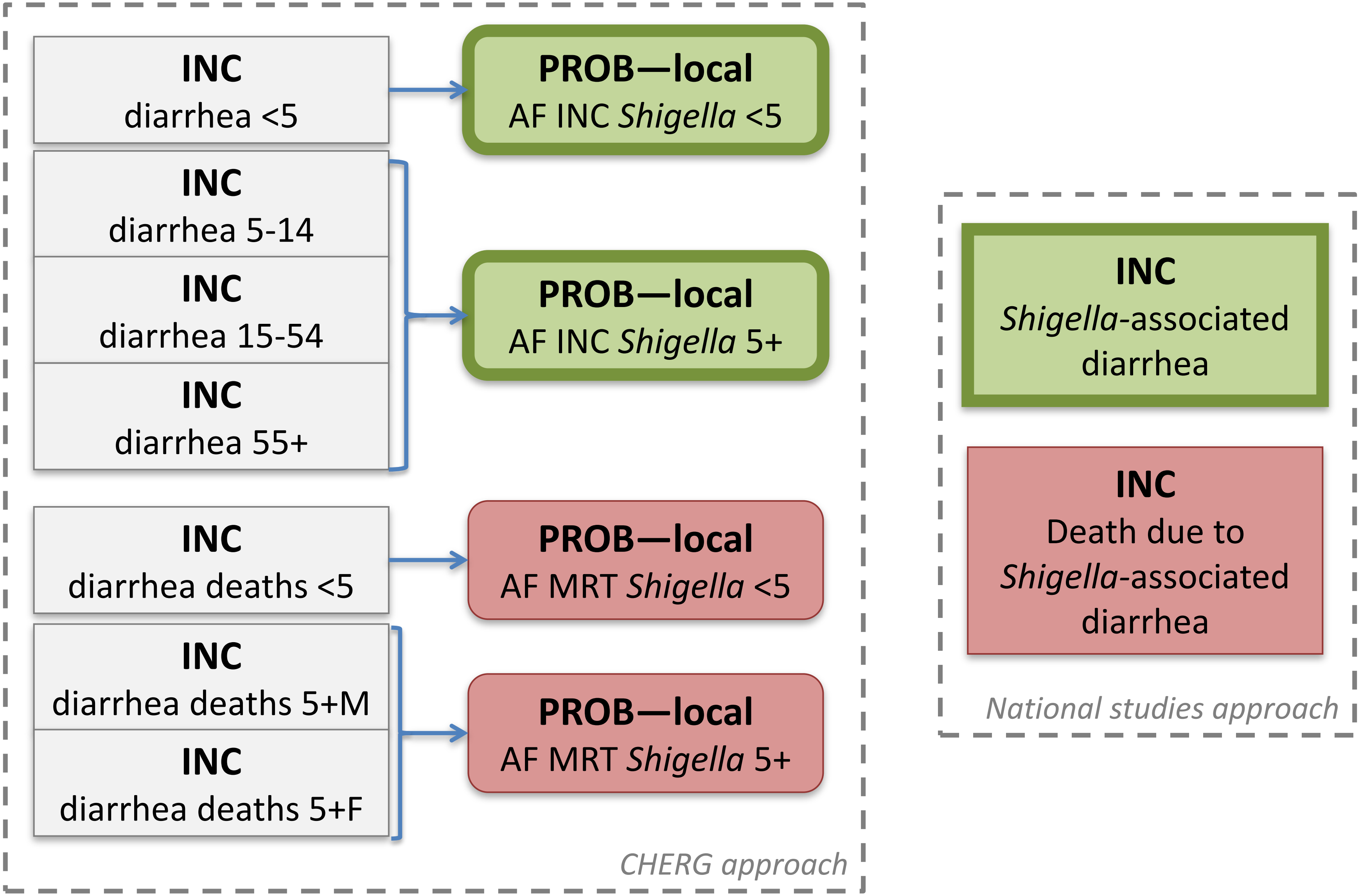

Vibrio cholerae
Disease Model

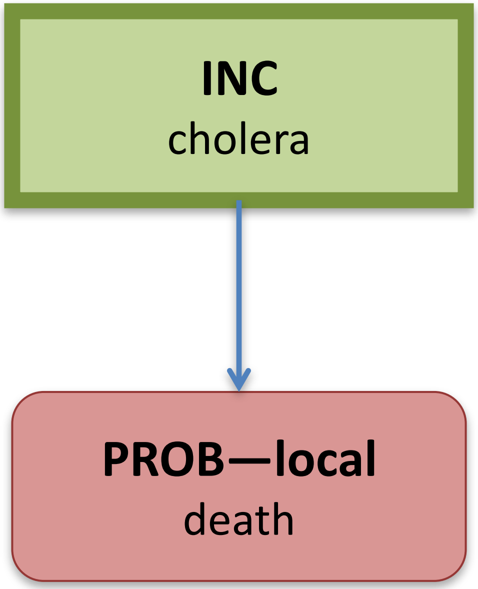

Cryptosporidium spp.
Disease Model

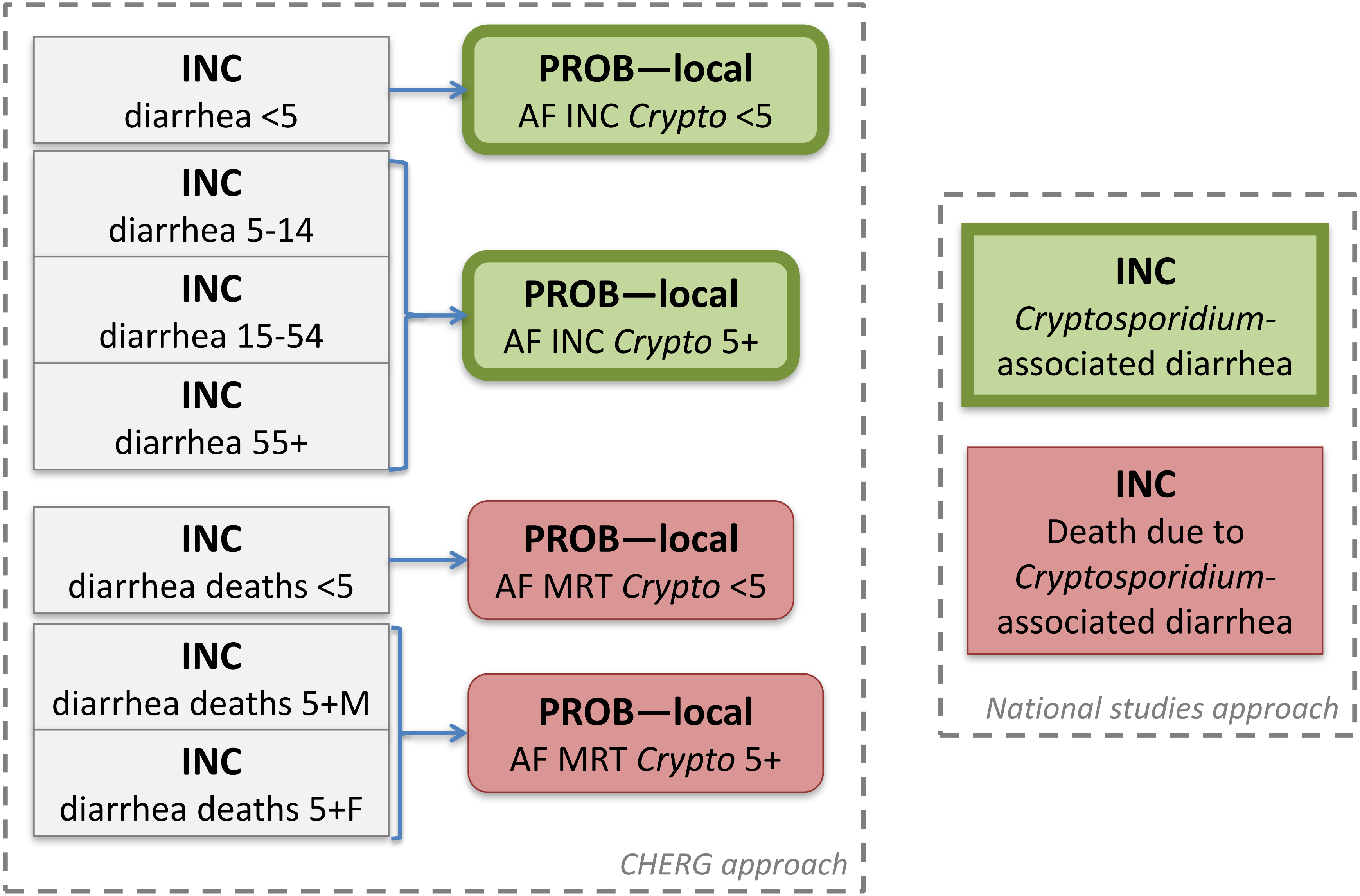

Entamoeba histolytica Disease Model

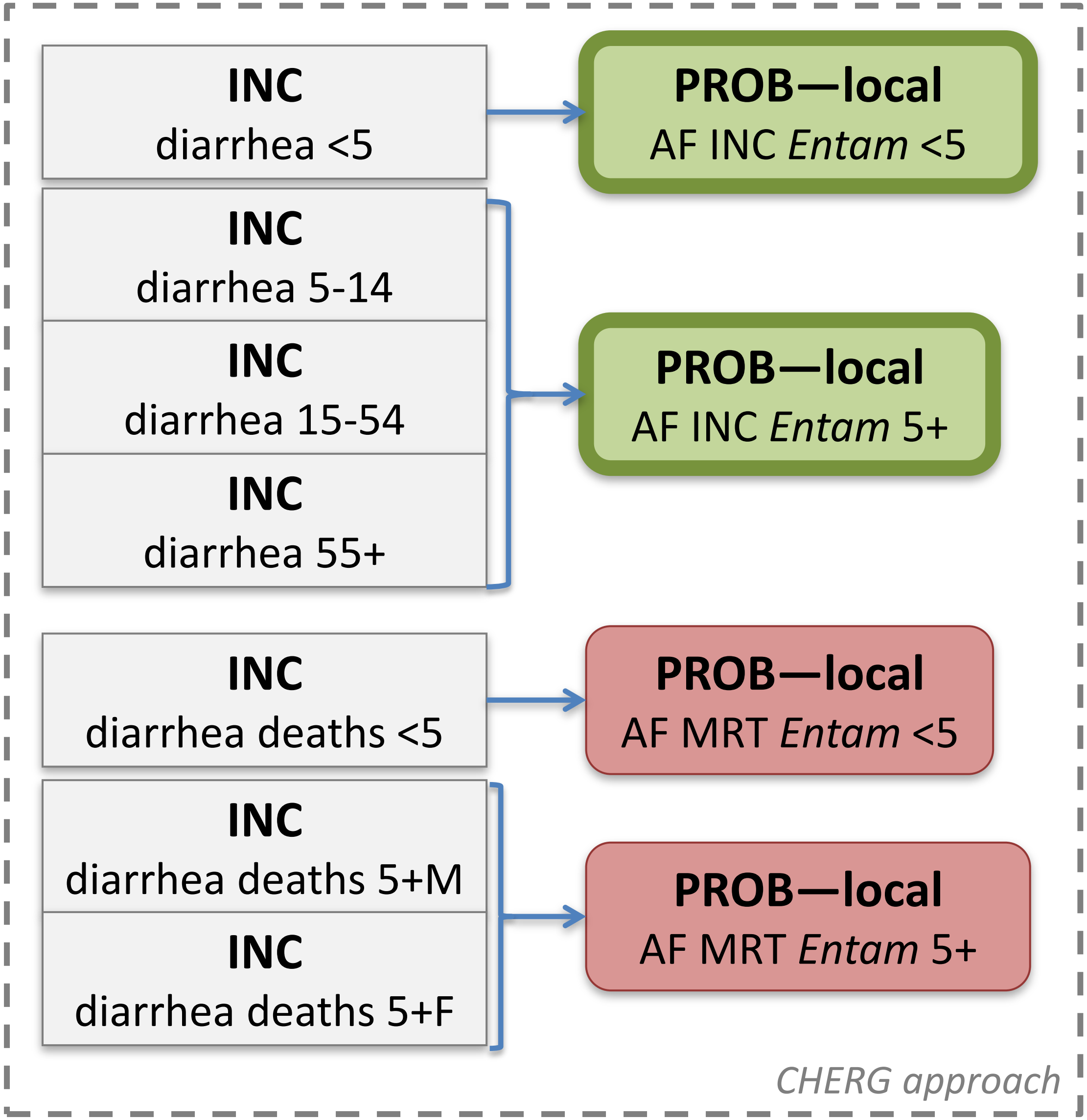

Giardia spp.
Disease Model

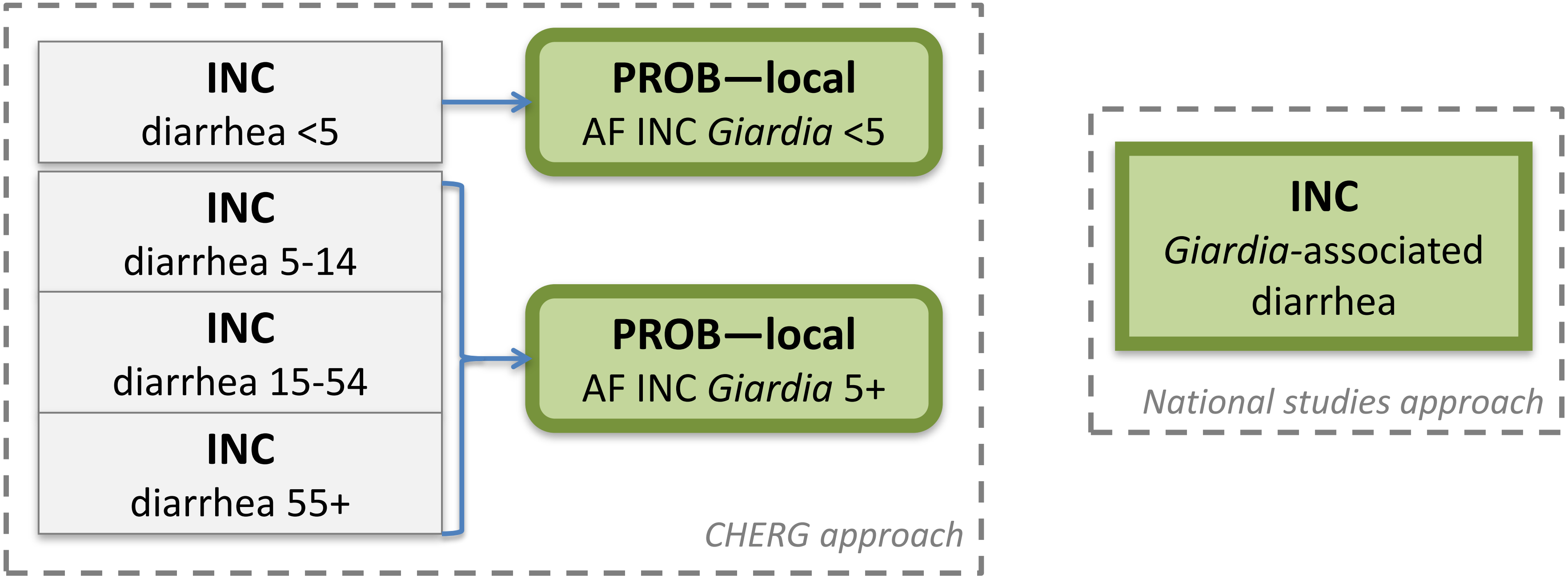

Hepatitis A virus
Disease Model

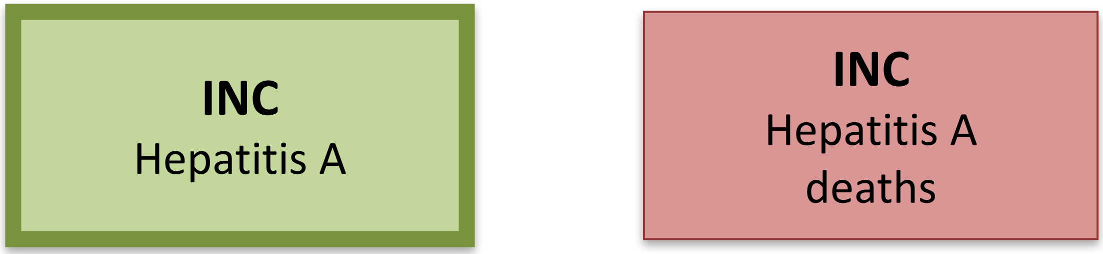

Brucella spp.
Disease Model

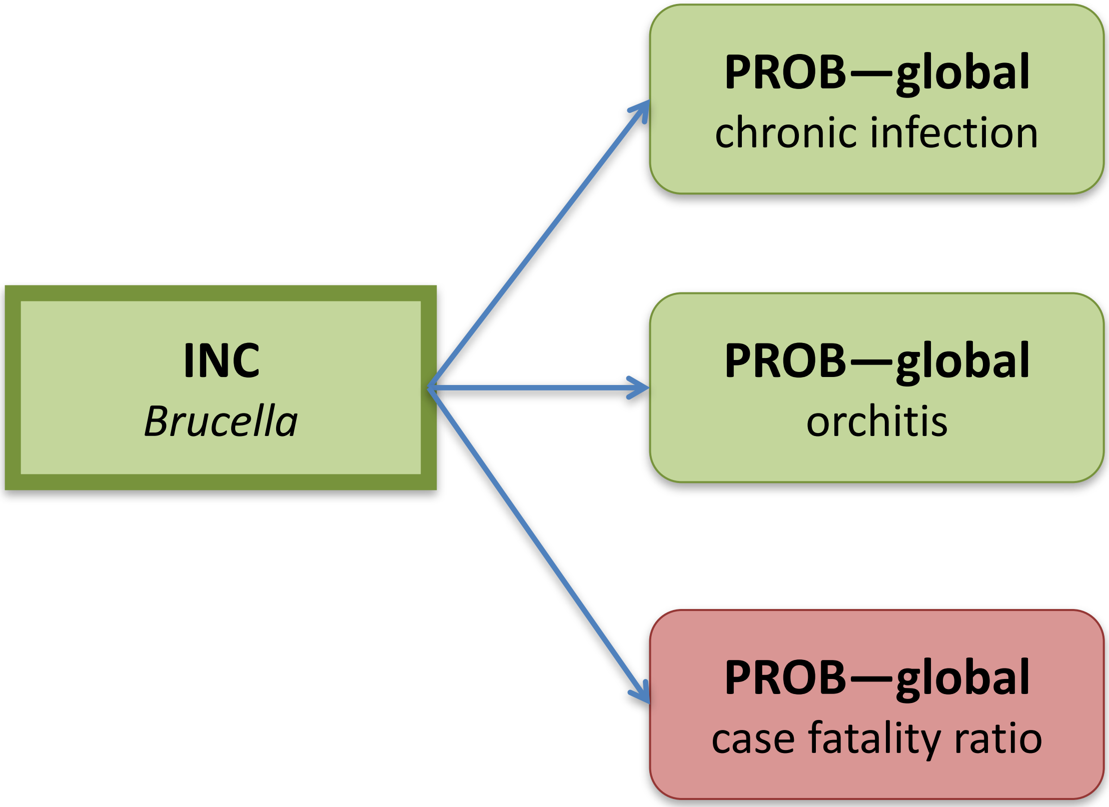

Listeria monocytogenes Disease Model

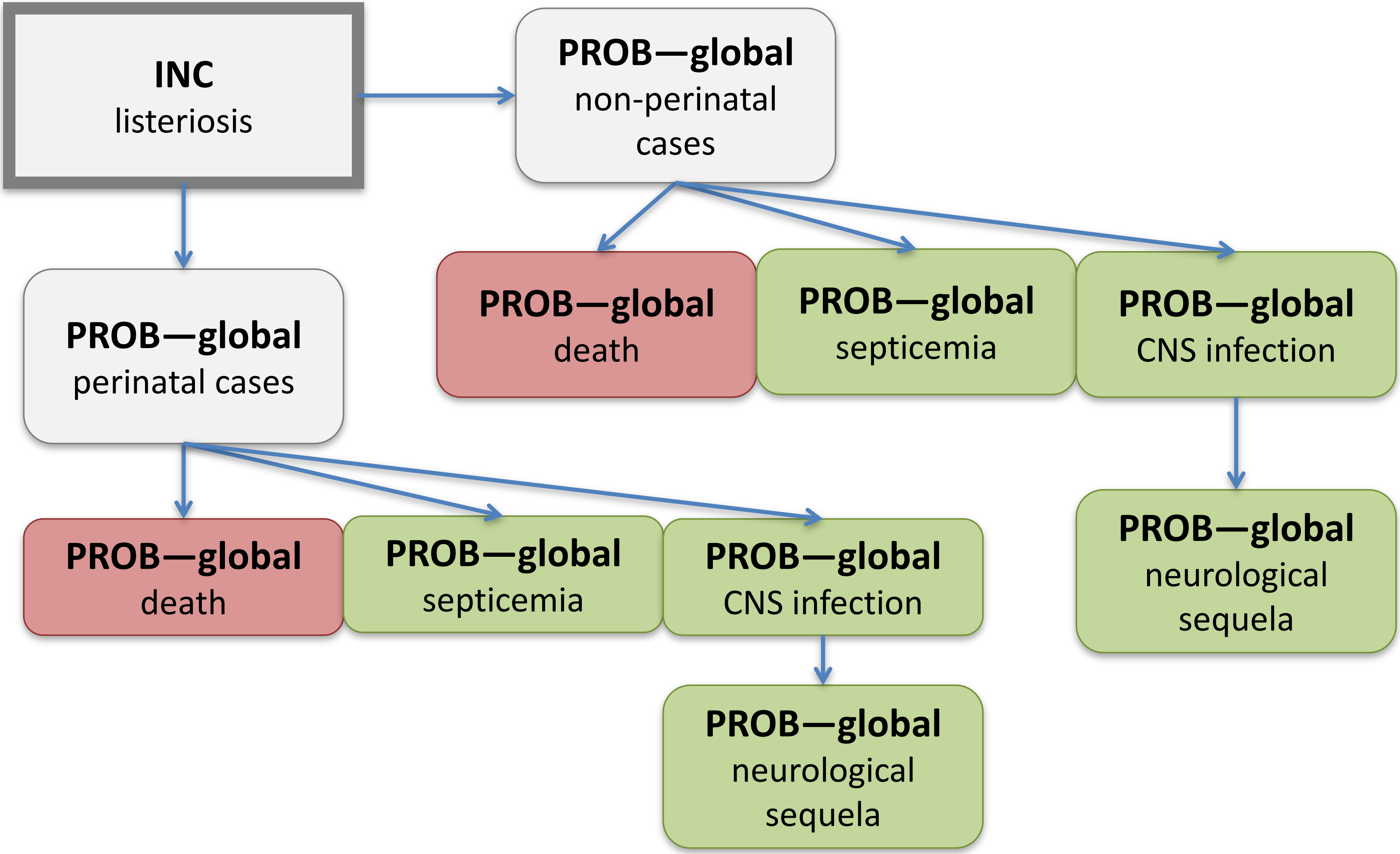

Mycobacterium bovis
Disease Model

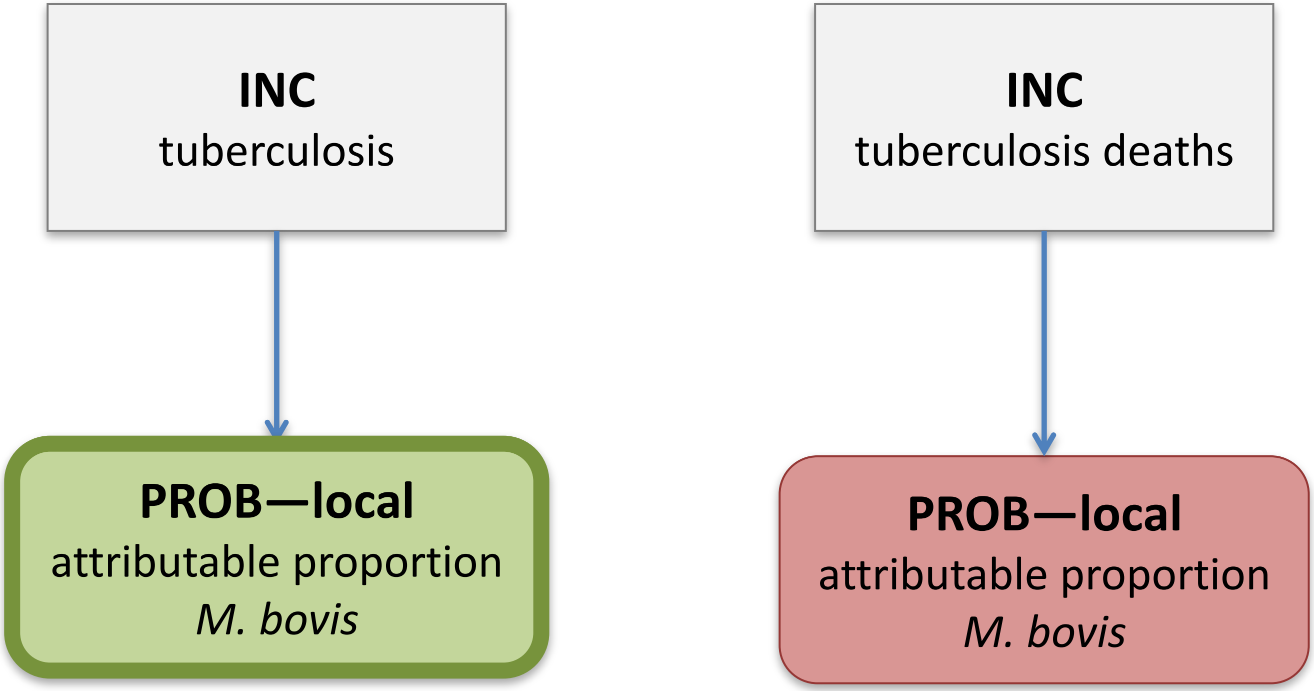

Salmonella **Paratyphi** Disease Model

INC
Paratyphoid fever

INC
Paratyphoid cysts

INC
Paratyphoid deaths

Salmonella **Typhi**
Disease Model

INC
Typhoid fever

INC
Typhoid cysts

INC
Typhoid deaths

Toxoplasma gondii (congenital) Disease Model

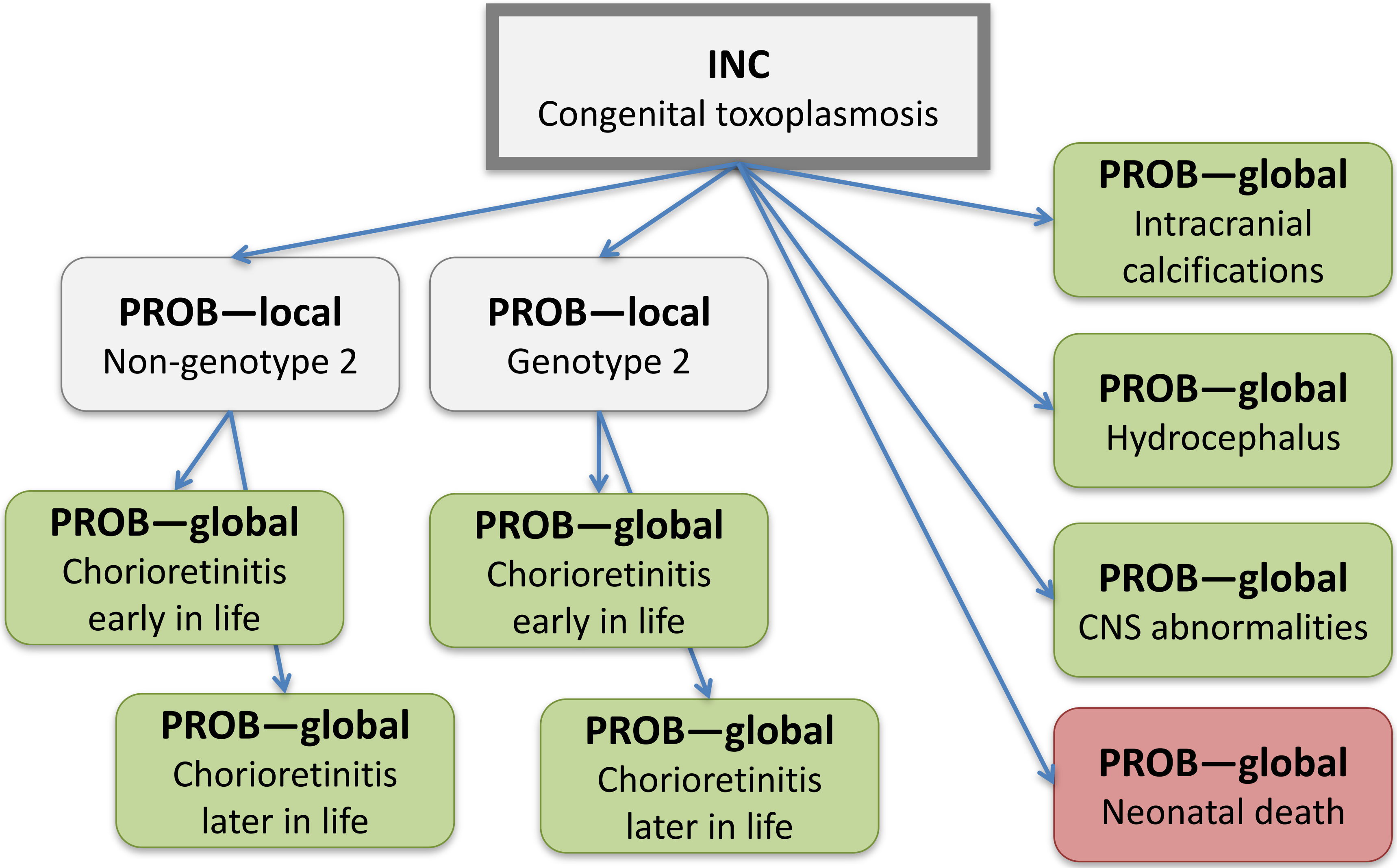

Toxoplasma gondii (acquired) Disease Model

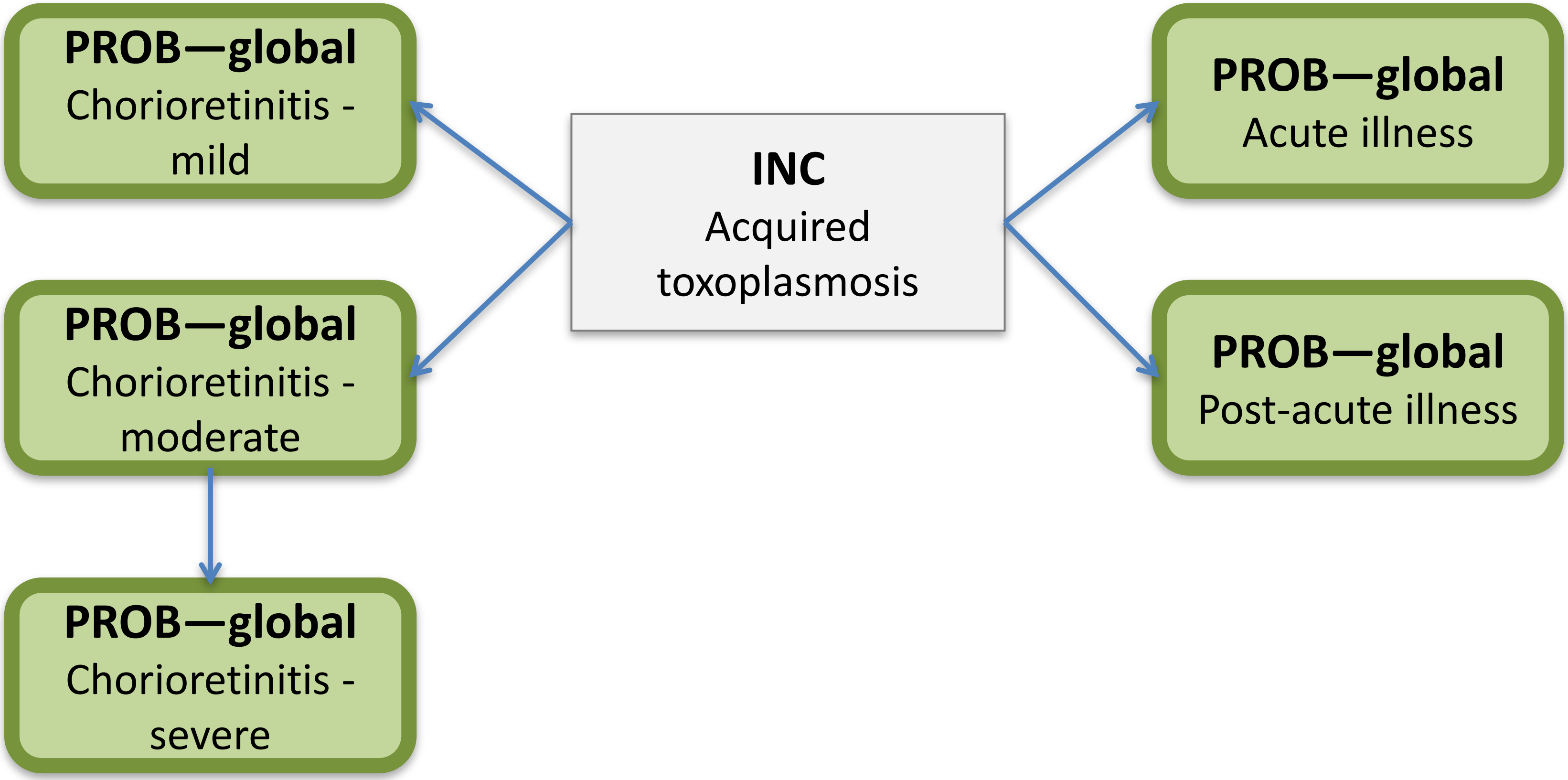

Bacillus cereus
Disease Model

INC
B. cereus

Clostridium botulinum
Disease Model

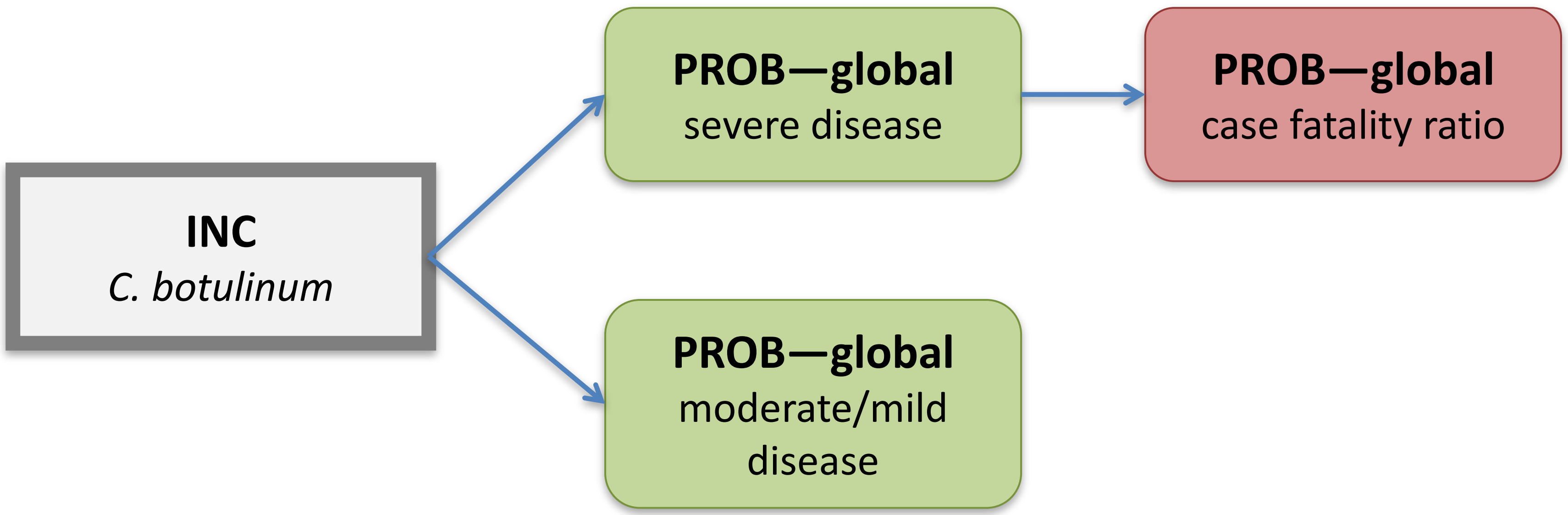

Clostridium perfringens Disease Model

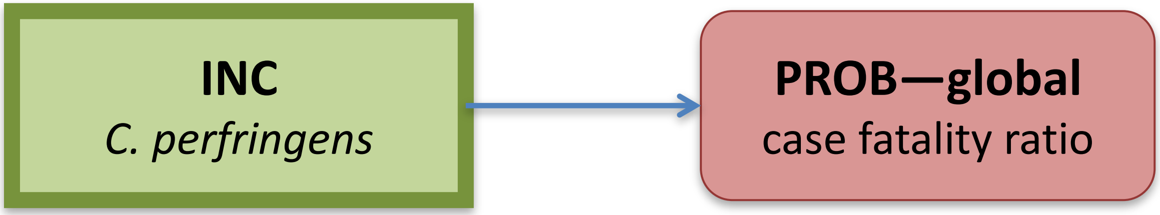

Staphylococcus aureus Disease Model

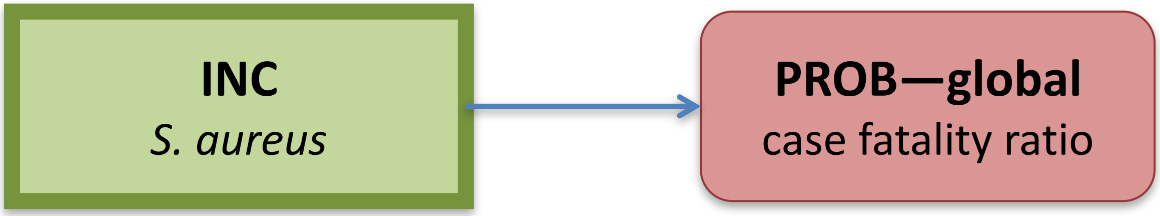

Echinococcus granulosus Disease Model

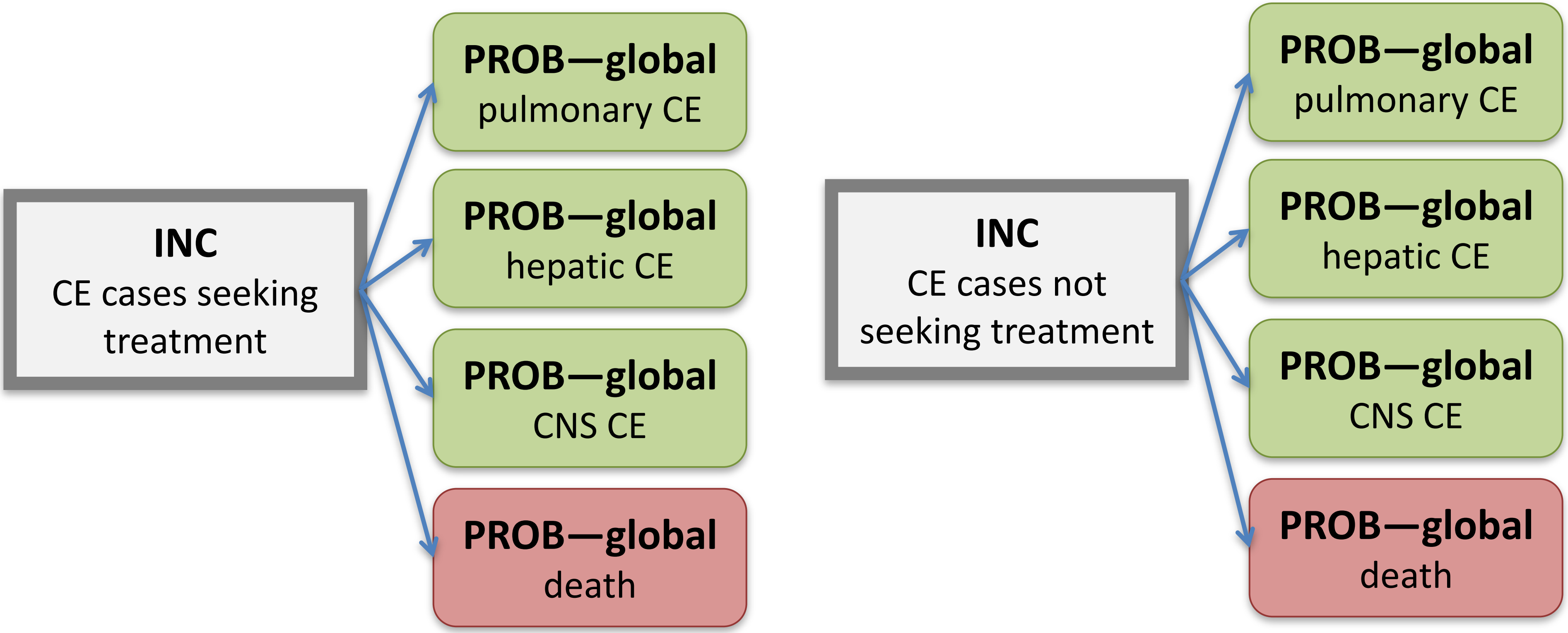

Echinococcus multilocularis Disease Model

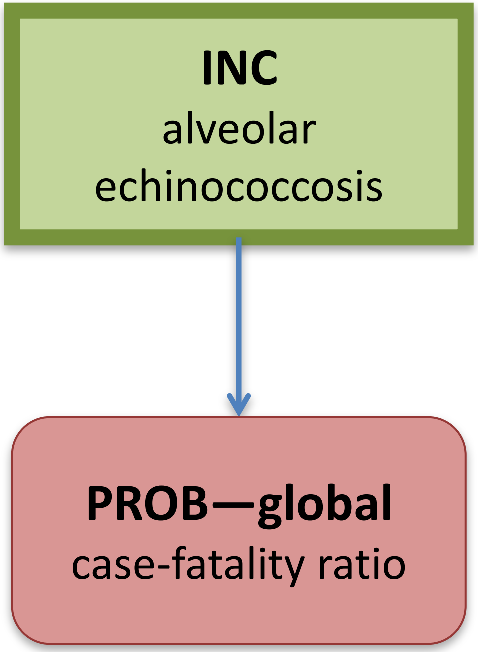

Taenia solium
Disease Model

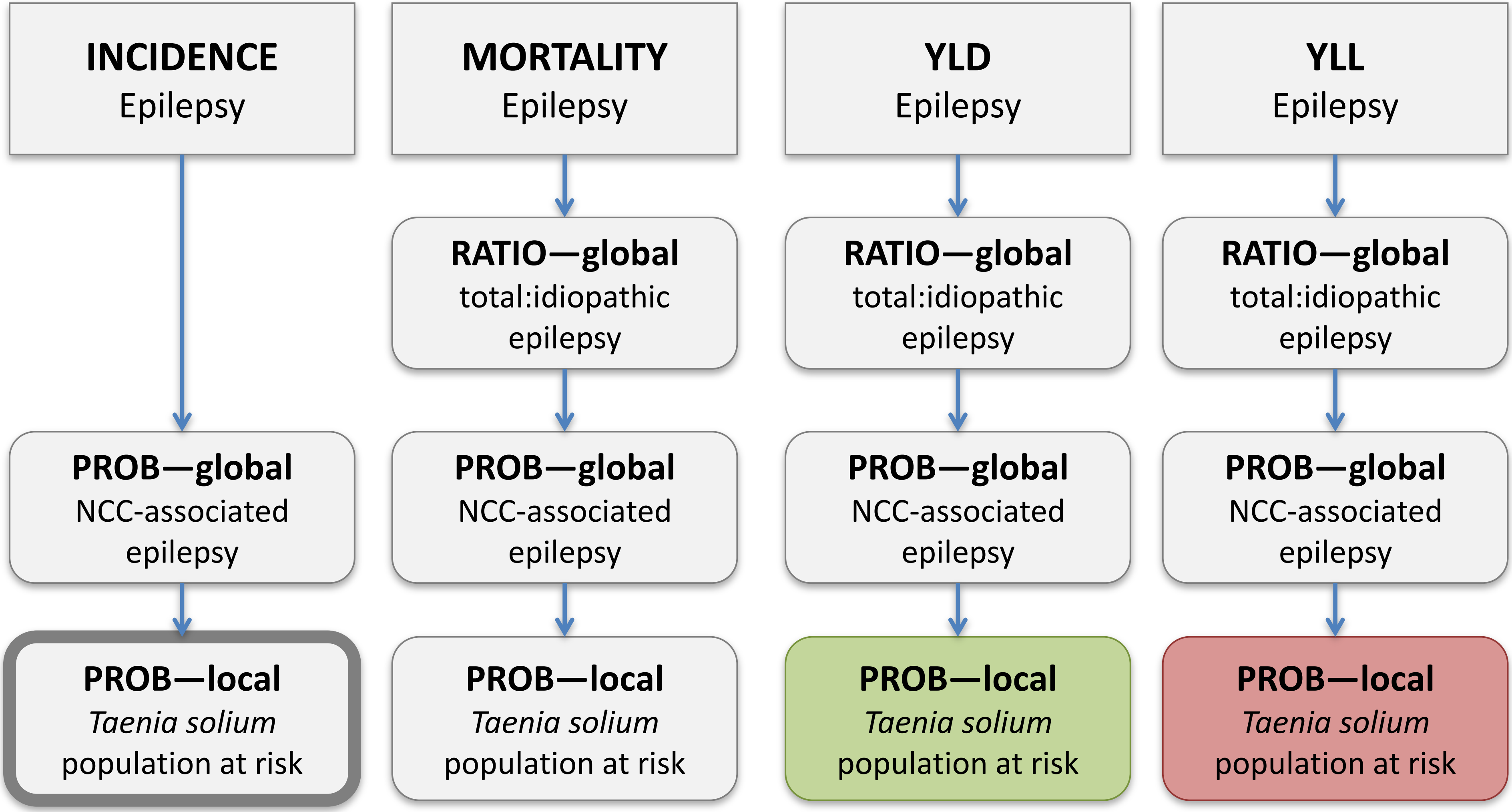

***Trichinella* spp.**
Disease Model

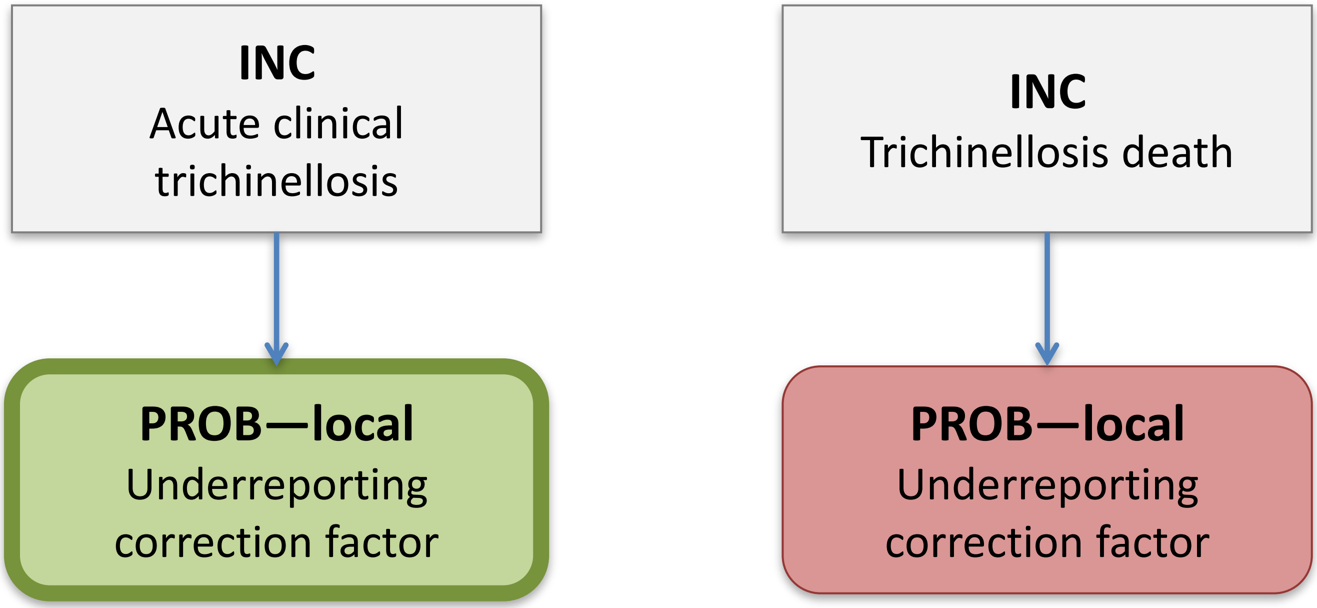

Ascaris spp.
Disease Model

INC
ascariosis

INC
ascariosis-related mild abdominopelvic problems

INC
ascariosis-related severe wasting

INC
ascariosis-related mortality

Clonorchis sinensis Disease Model

INC
symptomatic clonorchiosis

INC
clonorchiosis-related mortality

Fasciola spp.
Disease Model

INC
symptomatic
fasciolosis

Intestinal flukes
Disease Model

INC
symptomatic intestinal
trematodosis

Opisthorchis spp.
Disease Model

INC
symptomatic opisthorchiosis

INC
opisthorchiosis-related mortality

***Paragonimus* spp.**
Disease Model

**INC**<br>symptomatic paragonimosis	**INC**<br>cerebral paragonimosis	**INC**<br>paragonimosis-related mortality

Dioxin
Disease Model

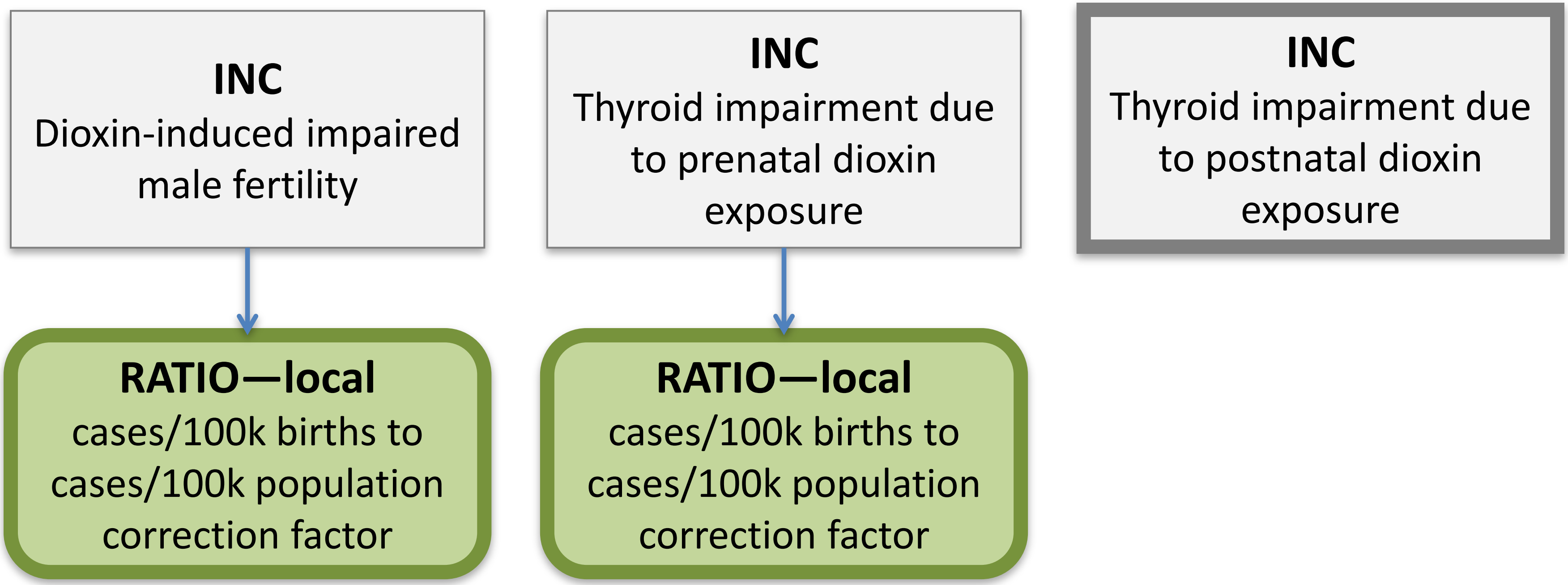

Aflatoxin
Disease Model

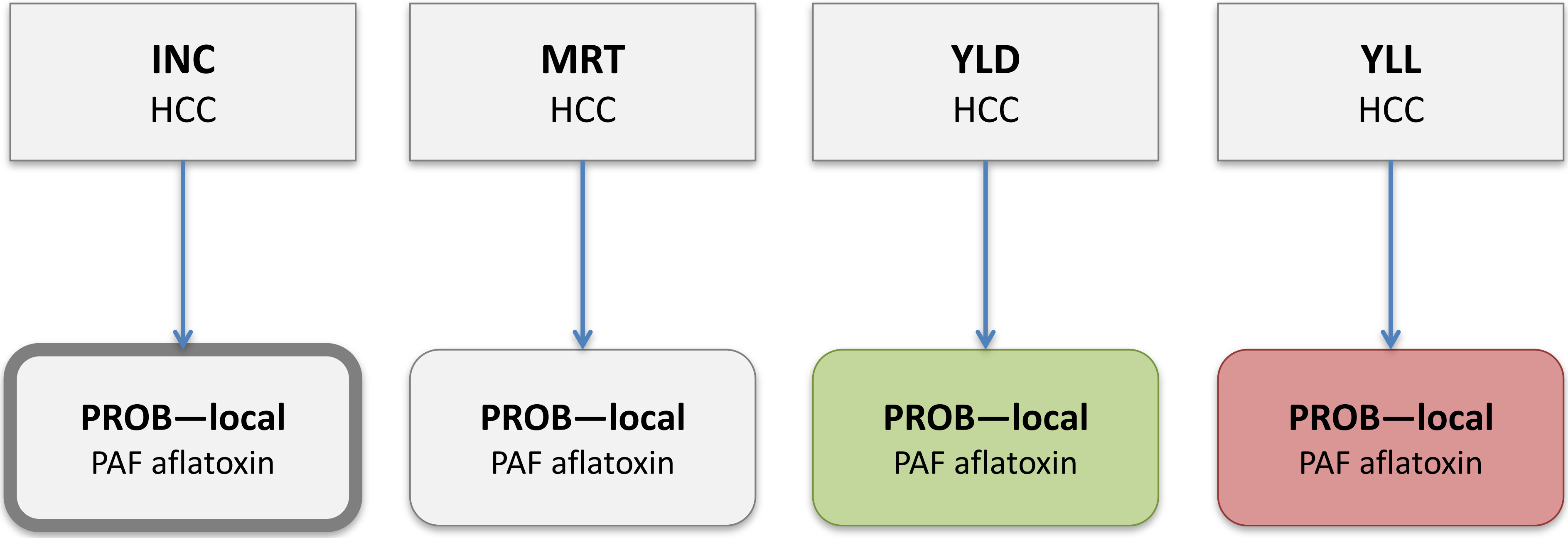

Cyanide in cassava
Disease Model

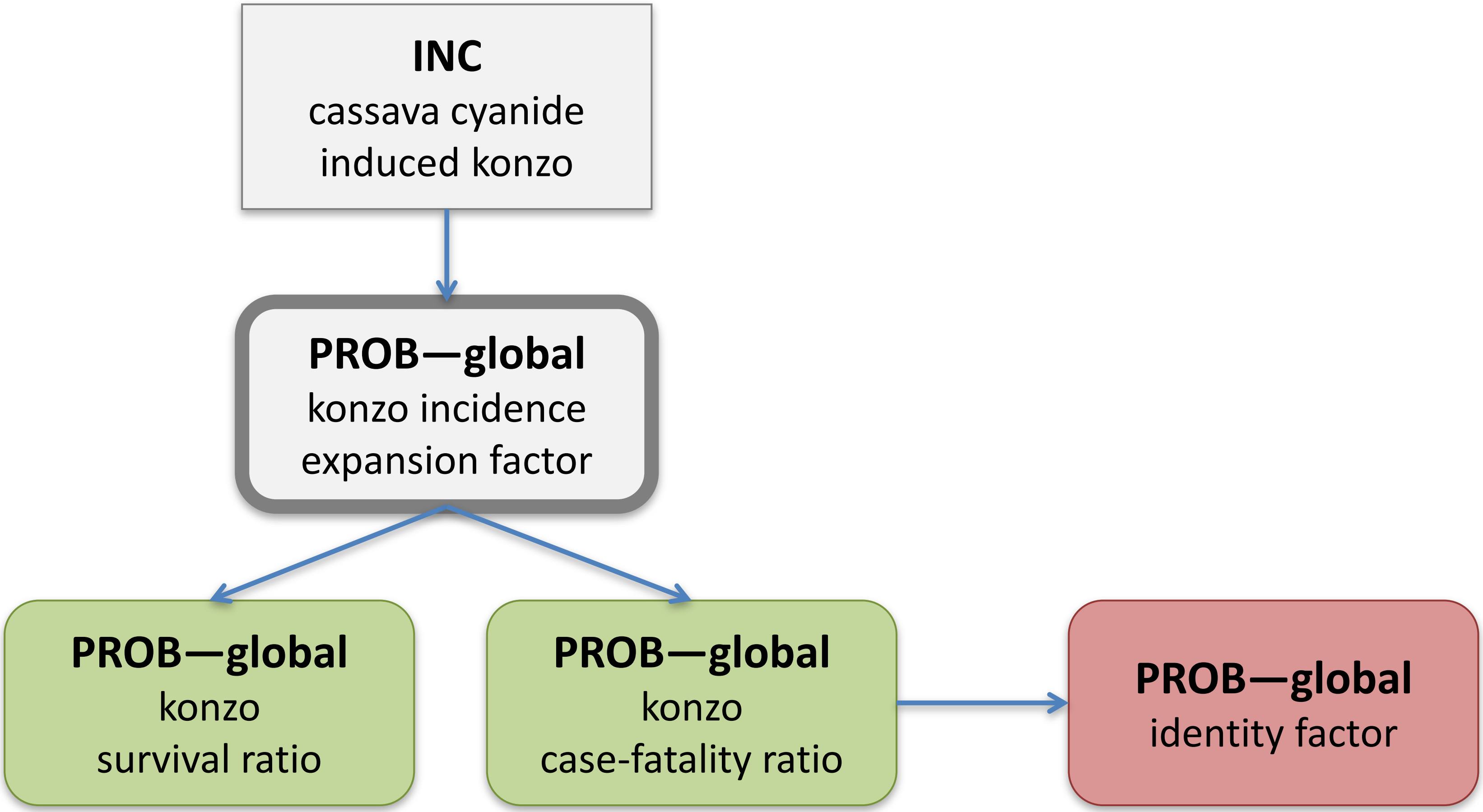

Peanut allergen
Disease Model

INC
Peanut-induced allergy

INC
Death due to peanut-induced allergy
