# Supplementary material for: Methodological Framework for World Health Organization Estimates of the Global Burden of Foodborne Disease
Source: PLoS One. 2015 Dec 3;10(12):e0142498. doi: 10.1371/journal.pone.0142498 (PMC4668830; doi:10.1371/journal.pone.0142498)
Supplement: S1 Table — (PDF) [file pone.0142498.s005.pdf]

S1 Table. Modelling strategies for the hazards included in the WHO global burden of foodborne disease estimates.

Hazard	Burden attribution approach	Disease model	Imputation	Foodborne attribution
Diarrheal disease agents				
Norovirus	Categorical attribution	Attributional	Pires et al. [1]	Expert elicitation [2]
Campylobacter spp.	Categorical attribution	Attributional	Pires et al. [1]	Expert elicitation [2]
Enteropathogenic *E. coli*	Categorical attribution	Attributional	Pires et al. [1]	Expert elicitation [2]
Enterotoxigenic *E. coli*	Categorical attribution	Attributional	Pires et al. [1]	Expert elicitation [2]
Shiga toxin-producing *E. coli*	Categorical attribution	Attributional	Pires et al. [1]	Expert elicitation [2]
Non-typhoidal *S. enterica*	Categorical attribution	Attributional	Pires et al. [1]	Expert elicitation [2]
Shigella spp.	Categorical attribution	Attributional	Pires et al. [1]	Expert elicitation [2]
Vibrio cholerae	Categorical attribution	Attributional	Pires et al. [1]	Expert elicitation [2]
Cryptosporidium spp.	Categorical attribution	Attributional	Pires et al. [1]	Expert elicitation [2]
Entamoeba histolytica	Categorical attribution	Attributional	Pires et al. [1]	Expert elicitation [2]
Giardia spp.	Categorical attribution	Attributional	Pires et al. [1]	Expert elicitation [2]
Invasive infectious disease agents				
Hepatitis A Virus	Categorical attribution	Direct: GBD 2010 [3]	N/A[a]	Expert elicitation [2]
Brucella spp.	Categorical attribution	Transitional	Random effects	Expert elicitation [2]
Listeria monocytogenes, perinatal	Categorical attribution	Transitional	Random effects	100%
Listeria monocytogenes, acquired	Categorical attribution	Transitional	Random effects	100%
Mycobacterium bovis	Categorical attribution	Attributional	N/A[a]	100%
Salmonella Paratyphi	Categorical attribution	Direct: GBD 2010 [3]	N/A[a]	Expert elicitation [2]
Salmonella Typhi	Categorical attribution	Direct: GBD 2010 [3]	N/A[a]	Expert elicitation [2]

Hazard	Burden attribution approach	Disease model	Imputation	Foodborne attribution
Toxoplasma gondii, congenital	Categorical attribution	Transitional	Random effects	Expert elicitation [2]
Toxoplasma gondii, acquired	Categorical attribution	Transitional	Random effects	Expert elicitation [2]
Enteric intoxications				
Bacillus cereus[b]	Categorical attribution	Direct	Uniform	100%
Clostridium botulinum[b]	Categorical attribution	Direct	Uniform	100%
Clostridium perfringens[b]	Categorical attribution	Direct	Uniform	100%
Staphylococcus aureus[b]	Categorical attribution	Direct	Uniform	100%
Cestodes				
Echinococcus granulosus, cases seeking treatment	Categorical attribution	Transitional	Random effects	Expert elicitation [2]
Echinococcus granulosus, cases not seeking treatment	Categorical attribution	Transitional	Random effects	Expert elicitation [2]
Echinococcus multilocularis	Categorical attribution	Transitional	Random effects	Expert elicitation [2]
Taenia solium	Categorical attribution	Attributional	N/A[a]	100%
Nematodes				
Ascaris spp.	Categorical attribution	Direct: GBD 2010 [3]	Random effects	Expert elicitation [2]
Trichinella spp.	Categorical attribution	Direct	N/A[a]	100%
Trematodes				
Clonorchis sinensis	Categorical attribution	Direct	Random effects	100%
Fasciola spp.	Categorical attribution	Direct	Random effects	100%
Intestinal flukes[c]	Categorical attribution	Direct	Random effects	100%
Opisthorchis spp.	Categorical attribution	Direct	Random effects	100%
Paragonimus spp.	Categorical attribution	Direct	Random effects	100%

**Hazard**	**Burden attribution approach**	**Disease model**	**Imputation**	**Foodborne attribution**
Organic pollutants				
Dioxin	Risk assessment	Direct	Random effects	100%
Toxins and allergens				
Aflatoxin	Counterfactual analysis	Attributional	Random effects	100%
Cyanide in cassava	Categorical attribution	Direct	Uniform	100%
Peanut allergens[b]	Categorical attribution	Direct	Uniform	100%

[a] No imputation had to be performed because data were used that had already been imputed.

[b] Excluded from global burden assessments.

[c] Includes *Echinostoma* spp., *Fasciolopsis buski*, *Heterophyes* spp., *Metagonimus* spp. and other foodborne intestinal trematode species.

References

1. Pires SM, Fischer-Walker CL, Lanata CF, Devleesschauwer B, Hall AJ, et al. (submitted) Aetiology-specific estimates of the global and regional incidence and mortality of diarrhoeal diseases commonly transmitted through food.

2. Hald T, Aspinall W, Devleesschauwer B, Cooke R, Corrigan T, et al. (submitted) Estimates of the relative contributions to the burden of disease due to selected foodborne hazards: a World Health Organization expert elicitation.

3. Murray CJL, Vos T, Lozano R, Naghavi M, Flaxman AD, et al. (2012) Disability-adjusted life years (DALYs) for 291 diseases and injuries in 21 regions, 1990-2010: a systematic analysis for the Global Burden of Disease Study 2010. Lancet 380(9859): 2197-2223.
