# Supplementary material for: Methodological Framework for World Health Organization Estimates of the Global Burden of Foodborne Disease
Source: PLoS One. 2015 Dec 3;10(12):e0142498. doi: 10.1371/journal.pone.0142498 (PMC4668830; doi:10.1371/journal.pone.0142498)
Supplement: S2 Table — The fourth column describes how the various DWs were derived. The source for the DWs is the Global Burden of Disease 2010 Study, unless stated otherwise. (PDF) [file pone.0142498.s006.pdf]

S2 Table. FERG hazards, causally related health states and corresponding disability weights (DWs). The fourth column describes how the various DWs were derived. The source for the DWs is the Global Burden of Disease 2010 Study, unless stated otherwise.

Hazard	Health state	DW	Mapping
Diarrheal hazards			
Norovirus	Diarrheal disease	0.074	**Weighted average** of 91% *Diarrhoea: mild* (DW=0.061); 8.5% *Diarrhoea: moderate* (DW=0.202); and 0.5% *Diarrhoea: severe* (DW=0.281)
Campylobacter spp.	Diarrheal disease	0.101	**Weighted average** of 73% *Diarrhoea: mild* (DW=0.061); 25% *Diarrhoea: moderate* (DW=0.202); and 2% *Diarrhoea: severe* (DW=0.281)
	Guillain-Barré syndrome	0.445	**Proxy health state** of *Multiple sclerosis: moderate*
Enteropathogenic *E. coli*	Diarrheal disease	0.074	**Weighted average** of 91% *Diarrhoea: mild* (DW=0.061); 8.5% *Diarrhoea: moderate* (DW=0.202); and 0.5% *Diarrhoea: severe* (DW=0.281)
Enterotoxigenic *E. coli*	Diarrheal disease	0.074	**Weighted average** of 91% *Diarrhoea: mild* (DW=0.061); 8.5% *Diarrhoea: moderate* (DW=0.202); and 0.5% *Diarrhoea: severe* (DW=0.281)
Shiga toxin-producing *E. coli*	Diarrheal disease	0.091	**Weighted average** of 80% *Diarrhoea: mild* (DW=0.061); 18% *Diarrhoea: moderate* (DW=0.202); and 2% *Diarrhoea: severe* (DW=0.281)
	Hemolytic uremic syndrome	0.210	**Proxy health state** of *Infectious disease: acute episode, severe*
	End-stage renal disease	0.573	**Mapped health state** of *End-stage renal disease: on dialysis*
Non-typhoidal *S. enterica*	Diarrheal disease	0.101	**Weighted average** of 73 % *Diarrhoea: mild* (DW=0.061); 25% *Diarrhoea: moderate* (DW=0.202); and 2% *Diarrhoea: severe* (DW=0.281)
	Invasive salmonellosis	0.210	**Proxy health state** of *Infectious disease: acute episode, severe*

Hazard	Health state	DW	Mapping
Shigella spp.	Diarrheal disease	0.101	**Weighted average** of 73 % *Diarrhoea: mild* (DW=0.061); 25% *Diarrhoea: moderate* (DW=0.202); and 2% *Diarrhoea: severe* (DW=0.281)
Vibrio cholerae	Diarrheal disease	0.194	**Weighted average** of 25 % *Diarrhoea: mild* (DW=0.061); 40% *Diarrhoea: moderate* (DW=0.202); and 35% *Diarrhoea: severe* (DW=0.281)
Cryptosporidium spp.	Diarrheal disease	0.074	**Weighted average** of 91% *Diarrhoea: mild* (DW=0.061); 8.5% *Diarrhoea: moderate* (DW=0.202); and 0.5% *Diarrhoea: severe* (DW=0.281)
Entamoeba histolytica	Diarrheal disease	0.074	**Weighted average** of 91% *Diarrhoea: mild* (DW=0.061); 8.5% *Diarrhoea: moderate* (DW=0.202); and 0.5% *Diarrhoea: severe* (DW=0.281)
Giardia spp.	Diarrheal disease	0.074	**Weighted average** of 91% *Diarrhoea: mild* (DW=0.061); 8.5% *Diarrhoea: moderate* (DW=0.202); and 0.5% *Diarrhoea: severe* (DW=0.281)
Invasive enteric hazards			
Hepatitis A Virus	Hepatitis	0.108	**Weighted average** of 50% *Infectious disease: acute episode, mild* (DW=0.005); and 50% *Infectious disease: acute episode, severe* (DW=0.210)
Brucella spp.	Acute brucellosis	0.132	**Weighted average** of 50% *Infectious disease: acute episode, moderate* (DW=0.053); and 50% *Infectious disease: acute episode, severe* (DW=0.210)
	Chronic brucellosis	0.079	**Proxy health state** of *Musculoskeletal problems: legs, moderate*
	Orchitis	0.097	**Mapped health state** of *Epididymo-orchitis*
Listeria monocytogenes, perinatal	Sepsis	0.210	**Proxy health state** of *Infectious disease: acute episode, severe*
	Central nervous system infection	0.426	**Weighted average**; see [Maertens de Noordhout et al. 2014]
	Neurological sequelae	0.292	**Weighted average**; see [Maertens de Noordhout et al. 2014]

**Hazard**	**Health state**	**DW**	**Mapping**
Listeria monocytogenes, acquired	Sepsis	0.210	**Proxy health state** of *Infectious disease: acute episode, severe*
	Central nervous system infection	0.426	**Weighted average**; see [Maertens de Noordhout et al. 2014] for details
	Neurological sequelae	0.292	**Weighted average**; see [Maertens de Noordhout et al. 2014] for details
Mycobacterium bovis	Tuberculosis	0.331	**Mapped health state** of *Tuberculosis: without HIV infection*
Salmonella Paratyphi	Paratyphoid fever	0.210	**Proxy health state** of *Infectious disease: acute episode, severe*
	Liver abscesses and cysts	0.254	**Proxy health state** of *Infectious disease: post-acute consequences (fatigue, emotional lability, insomnia)*
Salmonella Typhi	Typhoid fever	0.210	**Proxy health state** of *Infectious disease: acute episode, severe*
	Liver abscesses and cysts	0.254	**Proxy health state** of *Infectious disease: post-acute consequences (fatigue, emotional lability, insomnia)*
Toxoplasma gondii, congenital	Intracranial calcification	0.010	**Proxy health state**; see [Havelaar et al. 2007] for details
	Hydrocephalus	0.360	**Weighted average**; see [Havelaar et al. 2007] for details
	Chorioretinitis, 1st year of life	0.033	**Proxy health state** of *Distance vision: moderate impairment*
	Chorioretinitis, later in life	0.033	**Proxy health state** of *Distance vision: moderate impairment*
	Central nervous system abnormalities	0.360	**Weighted average**; see [Havelaar et al. 2007] for details
Toxoplasma gondii, acquired	Chorioretinitis, mild	0.004	**Proxy health state** of *Distance vision: mild impairment*
	Chorioretinitis, moderate	0.033	**Proxy health state** of *Distance vision: moderate impairment*
	Chorioretinitis, severe	0.191	**Proxy health state** of *Distance vision: severe impairment*
	Acute illness	0.053	**Mapped health state** of *Infectious disease: acute episode, moderate*
	Post-acute illness	0.254	**Mapped health state** of *Infectious disease: post-acute consequences (fatigue, emotional lability, insomnia)*
Enteric intoxications			
Bacillus cereus[a]	Acute intoxication	0.061	**Proxy health state** of *Diarrhoea: mild*

Hazard	Health state	DW	Mapping
Clostridium botulinum[a]	Moderate/mild botulism	0.198	**Proxy health state** of *Multiple sclerosis: mild*
	Severe botulism	0.445	**Proxy health state** of *Multiple sclerosis: moderate*
Clostridium perfringens[a]	Acute intoxication	0.061	**Proxy health state** of *Diarrhoea: mild*
Staphylococcus aureus[a]	Acute intoxication	0.061	**Proxy health state** of *Diarrhoea: mild*
Cestodes			
Echinococcus granulosus, cases seeking treatment	Pulmonary cystic echinococcosis	0.192	**Proxy health state** of *COPD and other chronic respiratory diseases: moderate*
	Hepatic cystic echinococcosis	0.123	**Proxy health state** of *Abdominopelvic problem: moderate*
	Central nervous system cystic echinococcosis	0.221	**Proxy health state** of *Motor plus cognitive impairments: moderate*
Echinococcus granulosus, cases not seeking treatment	Pulmonary cystic echinococcosis	0.015	**Proxy health state** of *COPD and other chronic respiratory diseases: mild*
	Hepatic cystic echinococcosis	0.012	**Proxy health state** of *Abdominopelvic problem: mild*
	Central nervous system cystic echinococcosis	0.054	**Proxy health state** of *Motor plus cognitive impairments: mild*
Echinococcus multilocularis	Alveolar echinococcosis	0.123	**Proxy health state** of *Abdominopelvic problem: moderate*
Taenia solium	Epilepsy: treated, seizure free	0.072	**Mapped health state** of *Epilepsy: treated, seizure free*
	Epilepsy: treated, with recent seizures	0.319	**Mapped health state** of *Epilepsy: treated, with recent seizures*
	Epilepsy: severe	0.657	**Mapped health state** of *Epilepsy: severe*
	Epilepsy: untreated	0.420	**Mapped health state** of *Epilepsy: untreated*
Nematodes			
Ascaris spp.	Ascariasis infestation	0.030	**Mapped health state** of *Intestinal nematode infections: symptomatic*
	Mild abdominopelvic problems due to ascariasis	0.012	**Mapped health state** of *Abdominopelvic problem: mild*

Hazard	Health state	DW	Mapping
	Severe wasting due to ascariasis	0.127	**Mapped health state** of *Severe wasting*
Trichinella spp.	Acute clinical trichinellosis	0.637	**Aggregate** of *Diarrhoea: moderate* (DW = 0.202); *Disfigurement: level 2, with itch or pain* (DW = 0.187); *Musculoskeletal problems: generalised, moderate* (DW = 0.292); and *Infectious disease: acute episode, severe* (DW = 0.210) [Devleesschauwer et al. 2014]
Trematodes			
Clonorchis sinensis	Abdominopelvic problems due to heavy clonorchiosis	0.123	**Proxy health state** of *Abdominopelvic problem: moderate*
Fasciola hepatica	Abdominopelvic problems due to heavy fasciolosis	0.123	**Proxy health state** of *Abdominopelvic problem: moderate*
Intestinal flukes[b]	Abdominopelvic problems due to heavy intestinal fluke infections	0.123	**Proxy health state** of *Abdominopelvic problem: moderate*
Opisthorchis spp.	Abdominopelvic problems due to heavy opisthorchiosis	0.123	**Proxy health state** of *Abdominopelvic problem: moderate*
Paragonimus spp.	Central nervous system problems due to heavy paragonimosis	0.420	**Proxy health state** of *Epilepsy: untreated*
	Pulmonary problems due to heavy paragonimosis	0.132	**Proxy health state** of *Asthma: uncontrolled*
Organic pollutants			
Dioxin	Infertility	0.056[c]	**Mapped health state** of *Infertility: primary*
	Hypothyroidy due to prenatal exposure	0.019[d]	**Mapped health state** of *Hypothyroidy*
	Hypothyroidy due postnatal exposure	0.019[d]	**Mapped health state** of *Hypothyroidy*

Hazard	Health state	DW	Mapping
Toxins and allergens			
Aflatoxin	Hepatocellular carcinoma: diagnosis and primary therapy	0.294	**Mapped health state** of *Cancer: diagnosis and primary therapy*
	Hepatocellular carcinoma: metastatic	0.484	**Mapped health state** of *Cancer: metastatic*
	Hepatocellular carcinoma: terminal phase with medication	0.508	**Mapped health state** of *Cancer: terminal phase with medication*
	Hepatocellular carcinoma: terminal phase without medication	0.519	**Mapped health state** of *Cancer: terminal phase without medication*
Cyanide in cassava	Konzo	0.065	**Weighted average** of 63% *Motor impairment: mild* (DW=0.012); 27% *Motor impairment: moderate* (DW=0.076); and 10% *Motor impairment: severe* (DW=0.377)
Peanut allergens[a]	Living with peanut-induced allergy	0.012	**Weighted average** of 94% *Asthma: controlled* (DW=0.009); and 6% *Generic uncomplicated disease: anxiety about diagnosis* (DW=0.054)

[a] Excluded from global burden assessments.

[b] Includes *Echinostoma* spp., *Fasciolopsis buski*, *Heterophyes* spp., *Metagonimus* spp. and other foodborne intestinal trematode species.

[c] Value taken from the World Health Organization Global Health Estimates (WHO 2013); note the higher value compared to GBD 2010 (Salomon et al. 2012).

[d] Value taken from the GBD 2013 disability weights (Salomon et al. 2015).

References

1. de Noordhout CM, Devleesschauwer B, Angulo FJ, Verbeke G, Haagsma J, Kirk M et al. (2014) The global burden of listeriosis: a systematic review and meta-analysis. Lancet Infect Dis. doi: 10.1016/S1473-3099(14)70870-9.
2. Devleesschauwer B, Praet N, Speybroeck N, Torgerson PR, Haagsma JA, De Smet K, et al. (2014) The low global burden of trichinellosis: evidence and implications. Int J Parasitol. doi:10.1016/j.ijpara.2014.05.006.
3. Havelaar AH, Kemmeren JM, Kortbeek LM (2007) Disease burden of congenital toxoplasmosis. Clin Infect Dis 44(11): 1467-1474.
4. Salomon JA, Vos T, Hogan DR, Gagnon M, Naghavi M, Mokdad A, et al. (2012) Common values in assessing health outcomes from disease and injury: disability weights measurement study for the Global Burden of Disease Study 2010. Lancet 380(9859): 2129-2143. doi:10.1016/S0140-6736(12)61680-8.
5. Salomon JA, Haagsma JA, Davis A, Maertens de Noordhout C, Polinder S, Havelaar AH, et al. (2015) Disability weights for the Global Burden of Disease 2013 study. Lancet Glob Health 3(11): e712–e723.doi: 10.1016/S2214-109X(15)00069-8.
6. World Health Organization (2013) WHO methods and data sources for global burden of disease estimates 2000-2011. Global Health Estimates Technical Paper. WHO/HIS/HSI/GHE/2013.4. Available: http://www.who.int/healthinfo/statistics/GlobalDALYmethods_2000_2011.pdf. Accessed 13 April 2015.
